# Disexcitation in the ASH/RIM/ADL negative feedback circuit fine-tunes hyperosmotic sensation and avoidance in *Caenorhabditis elegans*

**DOI:** 10.3389/fnmol.2023.1101628

**Published:** 2023-03-15

**Authors:** Hui Liu, Jing-Jing Wu, Rong Li, Ping-Zhou Wang, Jia-Hao Huang, Yu Xu, Jia-Lu Zhao, Piao-Ping Wu, Si-Jia Li, Zheng-Xing Wu

**Affiliations:** Key Laboratory of Molecular Biophysics of Ministry of Education, Institute of Biophysics and Biochemistry, College of Life Science and Technology, Huazhong University of Science and Technology, Wuhan, China

**Keywords:** hyperosmotic avoidance, negative feedback circuit, disexcitation, tyramine, octopamine, *Caenorhabditis elegans*

## Abstract

Sensations, especially nociception, are tightly controlled and regulated by the central and peripheral nervous systems. Osmotic sensation and related physiological and behavioral reactions are essential for animal well-being and survival. In this study, we find that interaction between secondary nociceptive ADL and primary nociceptive ASH neurons upregulates *Caenorhabditis elegans* avoidance of the mild and medium hyperosmolality of 0.41 and 0.88 Osm but does not affect avoidance of high osmolality of 1.37 and 2.29 Osm. The interaction between ASH and ADL is actualized through a negative feedback circuit consisting of ASH, ADL, and RIM interneurons. In this circuit, hyperosmolality-sensitive ADL augments the ASH hyperosmotic response and animal hyperosmotic avoidance; RIM inhibits ADL and is excited by ASH; thus, ASH exciting RIM reduces ADL augmenting ASH. The neuronal signal integration modality in the circuit is disexcitation. In addition, ASH promotes hyperosmotic avoidance through ASH/RIC/AIY feedforward circuit. Finally, we find that in addition to ASH and ADL, multiple sensory neurons are involved in hyperosmotic sensation and avoidance behavior.

## Introduction

Animals regularly encounter, sense, and avoid noxious environmental stimuli, such as hyperosmotic and hypoosmotic stress (Culotti and Russell, 1978; Hilliard et al., 2005), excessive mechanical forces (Walker et al., 2009; Campbell et al., 2015), aversive smells (Li and Liberles, 2015; Duan et al., 2020), heavy metal ions (Sambongi et al., 1999; Guo et al., 2015; Wu et al., 2022), harmful pH (Sambongi et al., 2000; Spalthoff and Gopfert, 2016), alkaloids quinine (Hilliard et al., 2004; Voelker et al., 2019), extreme temperatures (Takeishi et al., 2020), etc. Environmental osmolality fluctuation is a universal challenge for organisms. The instability of extracellular osmolality is a universal stress faced by all organisms (Burg, 1995; Burg et al., 2007). Hyperosmolality and hypoosmolality disrupt proteostasis and cause protein aggregation and misfolding, resulting in detrimental effects on a cell’s physiology and function (Burg, 1995; Choe and Strange, 2008; Burkewitz et al., 2012). Intracellular and extracellular osmolality stability is essential for all organisms’ physiological homeostasis, health, and survival. The noxious osmotic pressure of environments is the main factor that disrupts the osmotic homeostasis in organisms. Organisms, including bacteria, yeasts, plants, and animals, use physiologic and behavioral responses to resist the noxious osmotic pressure (Thrasher et al., 1980; Zerbe and Robertson, 1983; Hohmann, 2002; Sewards and Sewards, 2003; Ciura and Bourque, 2006). Hyperosmolality is a nociceptive stimulus in animals. Animals sense and avoid it. The sensation of hyperosmolality is a form of nociception.

Sensations, especially nociception, are tightly controlled and regulated by the central and peripheral nervous systems (Basbaum et al., 2009; Baliki and Apkarian, 2015; Peirs and Seal, 2016). The sensory modulations at the level of sensory neurons are essential for animals to achieve direct and rapid regulation of sensations and behaviors (Collet et al., 1998; Root et al., 2011). Nociception, the sense of nociceptive stimuli with actual or potential tissue injuries, produces a diverse set of sensations, pain perceptions, emotions, and actions, including behavioral responses. Primary nociception and its modulations at the level of sensory neurons or the initial chain of sensory pathways provide more veridical and instantaneous information for animals to achieve rapid, more fine-tuned, and concentrated behavioral responses (Baliki and Apkarian, 2015; Guo et al., 2015; Liu et al., 2019; Wu et al., 2022). Peripheral circuitry modulations of nociception include: innocuous afferent fibers gating the transmission of nociceptive fibers *via* a relay of a peripheral inhibitory neuron as proposed by gate control theory (Melzack and Wall, 1965), non-nociceptive ASI (Amphid Single Cilium I) neurons reciprocally inhibiting nociceptive ASH (Amphid Single Cilium H; Guo et al., 2015), and secondary nociceptive ASK (Amphid Single Cilium K) neurons suppressing ASH activities by providing cGMP through gap junctions in the nematode *Caenorhabditis elegans* (*C. elegans*; Voelker et al., 2019; Wu et al., 2022). The molecular and circuital mechanisms of nociception, especially pain perception, have been extensively studied. However, the regulation of nociception needs to be better understood, and related studies are of paramount significance.

*Caenorhabditis elegans* is a favored model for neuroscience studies because of its compact nervous system and experimental tractability (de Bono and Maricq, 2005). The *C. elegans* hermaphrodite nervous system consists of just 302 neurons. Chemical and electrical synapses in the nervous system are 7,446, and the neuronal connectome is well-established (White et al., 1986; Cook et al., 2019). The challenge lies in understanding neural signal integration, dynamic modulation of circuitry activities, and underlying mechanisms. Naturally, *C. elegans* inhabits soil environments with varied osmolality. It senses and avoids hyperosmolality by generating avoidances immediately (Culotti and Russell, 1978; Yu et al., 2017). The animal provides an opportunity to investigate the molecular and circuital mechanisms underlying hyperosmotic avoidance behavior.

The main nociceptive ASH neurons in *C. elegans* respond with increased calcium levels to diverse aversive stimuli, including hyperosmolality, nose touch, heavy metal ions (such as copper ions), and volatile repellents (Bargmann et al., 1990; Kaplan and Horvitz, 1993; Sambongi et al., 1999; Hilliard et al., 2002, 2004, 2005; Bargmann, 2006; Guo et al., 2015; Wang et al., 2015; Wen et al., 2020; Wu et al., 2022). ASH senses and triggers avoidance responses of multimodal noxious stimuli. Upon noxious stimulation, it displays a robust ON (increases in cytoplasmic calcium due to depolarization that occurs when the concentration of the chemical cue increases) and a relatively minor OFF (increases in cytoplasmic calcium that occur when the concentration of the chemical cue decreases) Ca^2+^ responses (Ferkey et al., 2021). ASH leads to avoidance responses from noxious stimuli through synapses on the forward and backward command interneurons (White et al., 1986; Piggott et al., 2011). ASH detects hyperosmolality *via* the OSM-9 TRPV (Transient Receptor Potential Vanilloid) channel and triggers hyperosmotic avoidance (Colbert et al., 1997; Liedtke et al., 2003). The secondary nociceptive ADL (Amphid Dual Ciliated Ending L) neurons engage in avoiding heavy metals (Sambongi et al., 1999), SDS (Ketschek et al., 2004), and aversive odors (e.g., octanol; Troemel et al., 1995). ASH plays a major role in avoidance behaviors. In contrast, ADL plays minor roles only evident when ASH is missing (Bargmann et al., 1990; de Bono and Maricq, 2005). In the naturally occurring nematode *Pristionchus pacificus*, ablation of ADL results in a significantly reduced hyperosmotic avoidance of 2 M glycerol, suggesting that ADL, in addition to ASH, contributes to the osmotic sensation and avoidance (Srinivasan et al., 2008).

Here, we used a reverse genetic screen, genetic manipulation, quantitative behavior assay, *in vivo* Ca^2+^ imaging, and neuronal manipulation to study the circuital mechanism underlying hyperosmotic sensation and avoidance in *C. elegans*. We identify a negative feedback circuit consisting of ASH, RIM (Ring Interneuron M) interneurons, and ADL. The circuit osmolality-dependently and differently regulates *C. elegans* avoidance of mild, medium, and high osmolality. It upregulates and does not affect medium and high osmolality avoidance, respectively. Upon hyperosmotic stimulation, both the primary nociceptor ASH and secondary nociceptor ADL are excited; the excited ADL excites ASH by the signaling pathway of FLP (FMRF-Like Peptide)-4 and the NPR (NeuroPeptide Receptor)-5 receptor; RIM interneuron, is excited by ASH through glutamate/NMR (NMDA class glutamate Receptor)-2 signaling, inhibits ADL *via* the tyramine/TYRA (TYRAmine Receptor)-3 pathway. Thus, ASH exciting RIM reduces ADL exciting ASH. The neural signal integration modality in the circuit is disexcitation. The circuit functions to fine-tune hyperosmotic sensation and avoidance. Disexcitation is a newly found modality that establishes the homeostasis of pumping and 5-HT production in food-sensing ADFs under food supply and deprivation conditions (Liu et al., 2019). In addition, ASH upregulates hyperosmotic avoidance through a forward circuit consisting of ASH, RIC (Ring Interneuron C), and AIY (Anterior Interneuron Y) neurons. Finally, we find that in addition to ASH and ADL, other glutamatergic sensory neurons AQR (Anterior Q-cell Derived Receptor), ASE (Amphid Single Cilium E), ASG (Amphid Single Cilium G), AWC (Amphid Wing Neuron C), and PQR (Posterior Q-cell Derived Receptor), are required for normal hyperosmotic avoidance in *C. elegans*. However, the circuital mechanisms of these sensory neurons need further studies.

## Results

### Reciprocal modulations between sensory neurons ASH and ADL regulate hyperosmotic avoidance behavior

Ablating ADL differently affects avoidance of high osmolality by 2 M glycerol in nematode *Pristionchus pacificus* and *C. elegans.* ADL ablation in the former species leads to a significant reduction of hyperosmotic avoidance, less intensively than ablating both ASH and ADL. In contrast, ADL ablation in the latter species does not affect the avoidance behavior (Srinivasan et al., 2008). A possible explanation for this difference is that the two species may have varied intrinsic sensitivity to osmolality. The ADL functions in the sense and avoidance of hyperosmolality in *C. elegans* need study.

To evaluate the ADL’s role in the avoidance of different osmolality, we used the drop test shown in Supplementary Figure S1A to assay avoidance of varied osmolality shocks in wild-type (WT) N2 as a control and ADL-specific neurotransmission-ablated animals. Neuron-specific neurotransmission ablation was conducted by specific expression of neuronal toxin TeTx in tested neurons. TeTx, a light chain of tetanus toxin, is a specific protease of a vesicular SNARE protein synaptobrevin essential for vesicle fusion with the plasma membrane. It hydrolyzes synaptobrevin and blocks vesicle fusion with the plasma membrane and, thus, synaptic transmission (Schiavo et al., 1992). It is used to successfully eliminate neurotransmission in tested neurons (neurons::*TeTx* in short; Macosko et al., 2009; Guo et al., 2015; Liu et al., 2019; Wen et al., 2020; Wu et al., 2022). In the test, the M13 buffer and different solutions of varied osmolality were used as a control and stimuli, respectively. Solutes of glycerol (99%), sodium chloride (NaCl), fructose (99%), and sorbitol (98%) were put into the M13 buffer to obtain varied concentrations. The measured osmolality of different solutions was as follows. M13 buffer, 0.28 Osm; glycerol/M13 solutions: 0.1 M, 0.41 Osm; 0.5 M, 0.88 Osm; 1.0 M, 1.37 Osm; 2.0 M, 2.29 Osm; 0.25 M NaCl/M13, 0.77 Osm; 0.5 M fructose/M13, 0.87 Osm; 0.5 M sorbitol/M13, 0.90 Osm (Supplementary Figure S1B). The hyperosmotic avoidance responses indicated by the percentage or ratio in the WT N2 and ADL (*ver-2p*, 2.7 kb, ADL specific)::*TeTx* transgenic animals were positively related to the osmolality by glycerol/M13 solutions of varied concentrations (Supplementary Figure S1C). The relationship was well fitted by a Hill function, with a half maximal effect (ED_50_) 0.8614 and 0.9775 Osm in the WT and ADL::*TeTx* animals, respectively. The ADL neurotransmission elimination significantly reduced animal avoidance of mild and medium hyperosmolality of 0.41 and 0.88 Osm by 0.1 and 0.5 M glycerol/M13 solutions. However, it did not affect the avoidance of high osmolality of 1.37 and 2.29 Osm by 1 M or 2 M glycerol/M13 solutions, compared with its effect in the WT N2. The difference was most significant at 0.88 Osm. We thus used 0.5 M glycerol/M13 solution (0.88 Osm) for the following experiments in this study unless otherwise indicated. We further used 0.25 M NaCl/M13 (0.77 Osm), 0.5 M fructose/M13 (0.87 Osm), and 0.5 M sorbitol/M13 (0.90 Osm) solutions to validate the observed behavioral response to 0.8 Osm glycerol/M13 is hyperosmotic avoidance. As expected, WT N2 animal displayed similar hyperosmotic avoidance of these solutions (Supplementary Figure S1D), supporting the behavior is the avoidance of hyperosmolality, not of a given solute.

**Figure 1:**
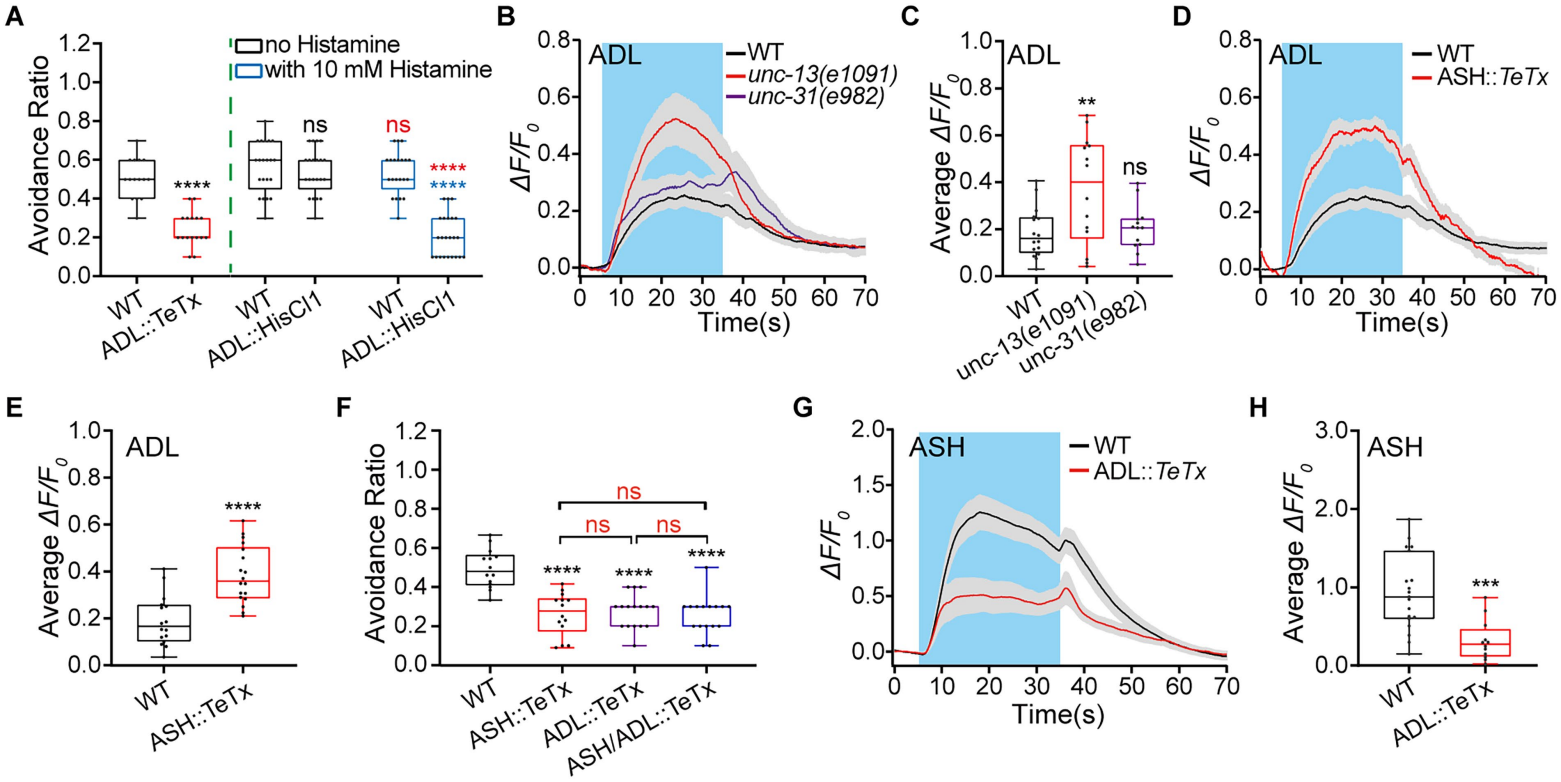
Interaction between ADL and ASH sensory neurons is required for normal avoidance of hyperosmolality in *C. elegans*. **(A)** The percentage or ratio of avoidance of a droplet of hyperosmolality by 0.88 Osm glycerol/M13 solution in wild-type (WT) N2 as control, ADL::*TeTx,* and ADL::*hisCl1* transgenic animals treated with or without 10 mM histamine. **(B–E)** The somal calcium signals in paired ADL sensory neurons in response to the hyperosmolality in WT, mutant, and transgenic animals of indicated genotypes. **(B,D)** Curves of Ca^2+^ transients presented as means (solid traces) ± SEM (gray shading) with cerulean background indicating the application of 0.88 Osm glycerol/M13 solution; **(C,E)** Box plots of the average intensity of Ca^2+^ signals of the ON response during glycerol/M13 solution perfusion with each dot representing the data from each individual tested animal. **(F)** Ratio of avoidance of the droplet of the hyperosmotic solution in the WT N2 and transgenic animals. **(G,H)** The somal calcium signals in ASH in response to the hyperosmolality in WT and ADL::*TeTx* transgenic worms. G, curves of Ca^2+^ transients presented as means (solid traces) ± SEM (gray shading) with cerulean background indicating the application of 0.88 Osm glycerol/M13 solution; H, box plots of the average intensity of Ca^2+^ transients of the ON response during glycerol/M13 solution perfusion with each dot representing the data from each tested animal. Heat maps of Ca^2+^ signals are shown in Supplementary Figure S1. Statistical significance is indicated as and in different colors: ns, not significant, ^**^p < 0.01, ^***^p < 0.001, and ^****^p < 0.0001; black, the tested vs. the WT; red, the histamine treated vs. the histamine untreated of the same genotype (in **A**) or as indicated (in **F**); blue, the tested vs. WT under histamine treatment (in **A**).

Continual inhibition of neurotransmission by TeTx beginning in embryonic periods may interfere with nervous system development. We next used chemogenetics to acutely inhibit the tested neurons and examine their neuronal functions in adult animals with HisCl1 channels and 10 mM histamine (neuron::chemogenetic inhibition, in short). HisCl1 is a histamine-gated chloride channel subunit from *Drosophila* that is effective for silencing neurons when activated by exogenous histamine (Pokala et al., 2014; Guo et al., 2015; Liu et al., 2019; Ge et al., 2020). Our results showed that ADL::*TeTx* and ADL::chemogenetic inhibition similarly reduced animals’ hyperosmotic avoidance (Figure 1A), supporting that ADL upregulates avoidance of medium hyperosmolality in *C. elegans*.

Given that ADL regulates hyperosmotic avoidance, it should respond to hyperosmotic stimulation cell-or non-cell-autonomously. We thus examined ADL calcium (Ca^2+^) responses to the medium hyperosmolality of 0.88 Osm by 0.5 M glycerol/M13 solution, using fluorescent Ca^2+^ imaging with GCaMP3.0 as a Ca^2+^ indicator (Tian et al., 2009) combined with microfluidic control of stimulation and animal movement (Chronis et al., 2007; Guo et al., 2015; Wang et al., 2015; Liu et al., 2019; Ge et al., 2020; Wen et al., 2020; Wu et al., 2022). ADL neurons in the WT N2 worm displayed robust Ca^2+^ responses to 0.88 Osm osmolality (Figures 1B,C; Supplementary Figure S1E). The changes in ADL Ca^2+^ signals may result from neurotransmission from other neurons. That is, ADL Ca^2+^ responses may be non-cell autonomous. We thus used *unc-13*(*e1091*) and *unc-31*(*e928*) mutant animals to assay the cell autonomy of the ADL hyperosmotic responses. *Unc-13* (UNCoordinated) encodes syntaxin-1 binding protein UNC-13, which is required for synaptic vesicle fusion with the presynaptic membrane and thus essential for neurotransmitter release (Richmond et al., 1999; Tokumaru and Augustine, 1999). The gene *unc-31* encodes UNC-31 protein, an ortholog of human CAPS (calcium-dependent secretion activator), which is essential for the exocytosis of dense cored vesicles and thus the release of neuropeptides (Avery et al., 1993; Lin et al., 2010). In *unc-13* or/and *unc-31* mutant animal/s, if the sensory response of a tested sensory neuron is significantly reduced or even disappears, then the neuronal response is non-cell autonomous; if the response remains unchanged, then it is cell autonomous; if the response increases, then it is cell autonomous and is inhibited by other neuron/s. Our result showed that *unc-13* and *unc-31* mutant animals displayed significantly augmented and WT ADL Ca^2+^ responses, respectively (Figures 1B,C; Supplementary Figure S1E). This result indicates that ADL Ca^2+^ responses to hyperosmolality are cell-autonomous and may be inhibited by neurotransmission mediated by classical neurotransmitter/s from other neurons.

ASH is the known main hyperosmolality-sensitive neuron so it may be the source of ADL inhibition. We then employed ASH::*TeTx* transgenic animals to determine the ASH source of ADL inhibition. ASH-specific expression of TeTx was driven by the *srv-11* promoter (1.9 kb upstream of the start codon; Taniguchi et al., 2014; O'Donnell et al., 2020). The ASH::*TeTx* transgenic animal displayed significantly increased ADL Ca^2+^ responses to 0.88 Osm osmolality, similar to *unc-13*(*e1091*) animal (Figures 1D,E; Supplementary Figures S1F,G). The result suggests that ASH inhibits the ADL hyperosmotic sensory response.

Given that ASH and ADL are primary and secondary hyperosmolality-sensitive neurons. Genetically ablating neurotransmission in these two types of neurons should display different impacts on the animal’s hyperosmotic avoidance behavior. We thus examined the hyperosmotic avoidance in transgenic animals of ASH::*TeTx*, ADL::*TeTx*, and ASH/ADL::*TeTx*. The expression of TeTx in ASH and ADL was directed by promoters *srv-11p* (in ASH), *ver-2p* (2.7 kb, in ADL), and *gap-11p* (3.3 kb, in ASH and ADL), respectively. Interestingly, genetic inhibition of neurotransmission in ADL, ASH, and ADL/ASH neurons similarly reduced the hyperosmotic avoidance of 0.88 Osm hyperosmolality by glycerol/M13 solution in transgenic animals (Figure 1F) in the wet drop test. This suggests that ASH and ADL may function in the same pathway and form a feedback circuit to regulate hyperosmotic avoidance. Based on the above results, ASH inhibits ADL Ca^2+^ responses to the medium hyperosmolality of 0.88 Osm. ADL in a possible feedback circuit may upregulate ASH Ca^2+^ responses to hyperosmolality. Indeed, genetically eliminating ADL neurotransmission by TeTx reduced ASH somal Ca^2+^ transients in response to the 0.88 Osm hyperosmolality stimulation (Figures 1G,H; Supplementary Figure S1H), suggesting that ADL excites ASH.

The above results suggest that an interaction between ASH and ADL sensory neurons regulates hyperosmotic sensation and avoidance behavior in *C. elegans*. In this interaction, ADL excites ASH; in contrast, ASH inhibits ADL.

### ADL excites ASH *via* the FLP-4/NPR-5 signaling pathway

**Figure 2:**
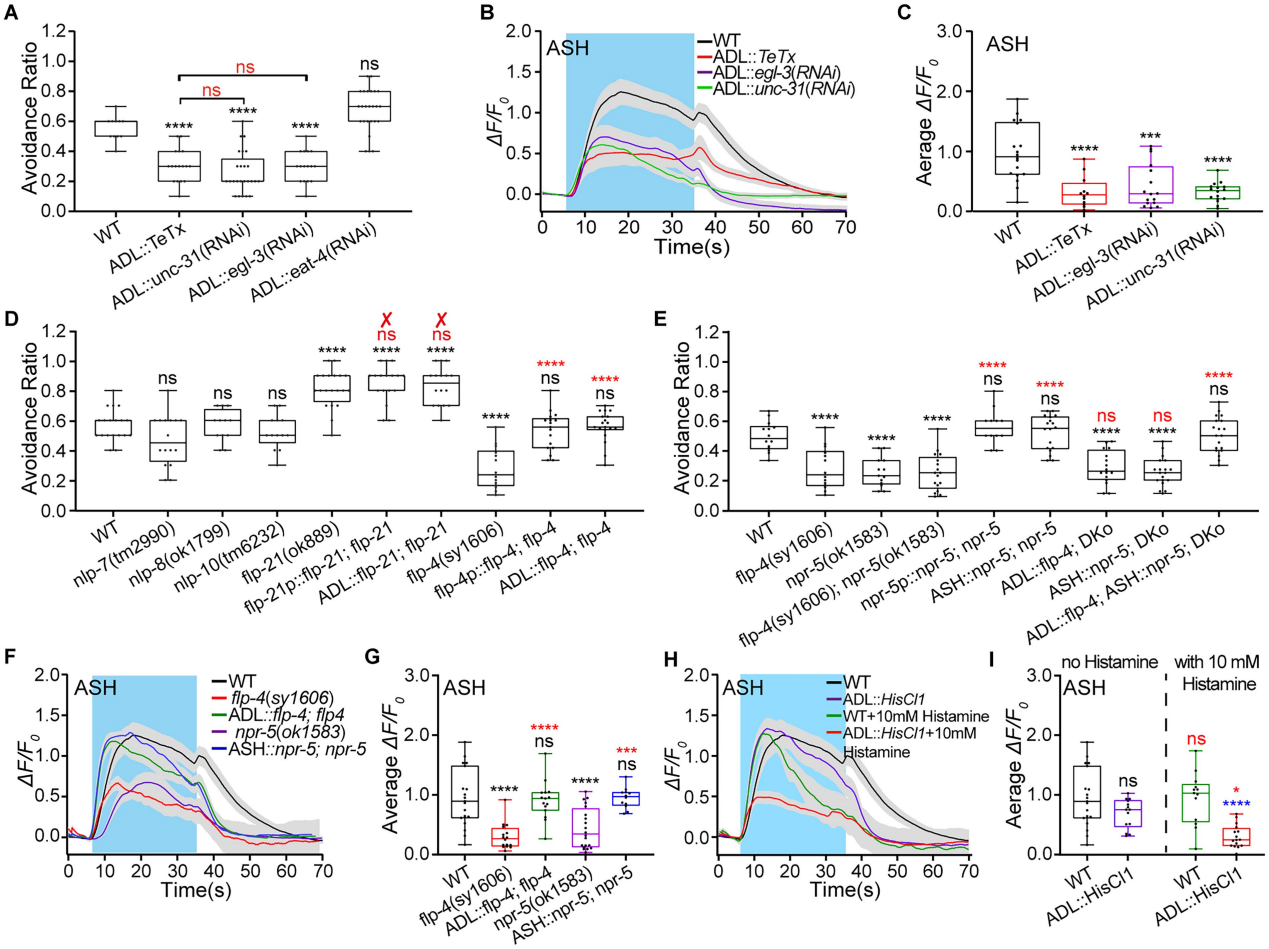
ADL excites ASH *via* the FLP-4/NPR-5 signaling pathway to promote animal avoidance of 0.88 Osm hyperosmolality. **(A)**. Ratio of avoidance of a droplet of the 0.88 Osm glycerol/M13 hyperosmotic solution in wild-type (WT) N2 (as a control) and transgenic animals of indicated genotypes. **(B,C)** The somal calcium transients in paired ASH sensory neurons responding to the hyperosmolality in WT N2 and transgenic animals of indicated genotypes. **(B)** Curves of Ca^2+^ transients presented as means (solid traces) ± SEM (gray shading) with cerulean background indicating the application of 0.88 Osm glycerol/M13 solution; **(C)** Box plots of the average intensity of Ca^2+^ signals of the ON response during glycerol/M13 solution perfusion with each dot representing the data from each tested worm. **(D,E)** Ratio of animals’ avoidance of a droplet of the hyperosmotic solution in animals of indicated genotypes. “✘” means the failure to restore WT behavior by the genetic rescue indicated. **(F,G)** The somal calcium transients in paired ASH sensory neurons in response to the application of the hyperosmolality in animals of indicated genotypes. **(F,H)** Curves of Ca^2+^ transients presented as means (solid traces) ± SEM (gray shading) with cerulean background indicating the application of 0.88 Osm glycerol/M13 solution; **(G,I)** Box plots of the average intensity of Ca^2+^ signals of the ON response during glycerol/M13 solution perfusion with each dot representing the data from each tested animal. Heat maps of Ca^2+^ signals are shown in Supplementary Figure S2. Statistical significance is indicated as and in different colors: ns, not significant, ^*^p < 0.05, ^***^p < 0.001, and ^****^p < 0.0001; black, the tested vs. the WT; red, the gene rescued vs. the related mutant, or the histamine treated vs. the histamine untreated of the same genotype (in **I**); blue, the tested vs. WT under histamine treatment (in **I**).

What is the mechanism for ADL exciting ASH? ADL is glutamatergic, neuropeptidergic, and presynaptic to ASH (White et al., 1986; Li and Kim, 2008; Cook et al., 2019). ASH expresses neuropeptidergic receptors NPR-1, a receptor for FMRF-Like peptides FLP-18 and FLP-21, but not known glutamatergic receptors.^1^ Thus, ADL likely excites ASH by neuropeptide signaling. We first used *egl-3* and *unc-31* knocked-down animals by ADL-specific RNA interference (RNAi) to identify the role of neuropeptide signaling in ADL regulating ASH. The *egl-3* gene encodes a homolog of mammalian proprotein convertase EGL (EGg Laying defective)-3 essential for neuropeptides biosynthesis (Li and Kim, 2008; Nkambeu et al., 2019). The *unc-31* is necessary for neuropeptide release from dense cored vesicles (Avery et al., 1993; Lin et al., 2010). As expected, ADL-specific RNAi knockdown of *unc-31* and *egl-3* significantly reduced the avoidance behavior as strongly as TeTx-mediated ablation of ADL neurotransmission (Figure 2A). We then used Ca^2+^ imaging to examine the effects of ADL::*TeTx,* ADL::*unc-31*(RNAi), and ADL::*egl-3*(RNAi) genetic manipulations on ASH Ca^2+^ responses to the 0.88 Osm stimulation. ADL::*TeTx,* ADL::*unc-31*(RNAi), and ADL::*egl-3*(RNAi) similarly decreased ASH Ca^2+^ responses to the hyperosmolality (Figures 2B,C; Supplementary Figure S2A). The data suggest that neuropeptide/s released from ADL mediate/s ADL excitation of ASH. ADL expresses FLP-4, FLP-21, neuropeptide-like peptides NLP-7, NLP-8, and NLP-10 (see footnote 1; Li and Kim, 2008). Thus, we screened the neuropeptide/s involved in the hyperosmotic avoidance regulation and the interaction between ADL and ASH, using *flp-4*, *flp-21*, *nlp-7*, *nlp-8*, and *nlp-10* mutant. Among these mutant animals, only the *flp-4* mutant animal displayed significantly reduced hyperosmotic avoidance compared with the WT N2. The *flp-4* genetic rescue expression in its expression neurons and ADL alone, driven by the *flp-4* promoter and *ver-2* promoter, fully restored the WT behavioral phenotype (Figure 2D). In contrast, *flp-21*(*ok889*) mutant showed augmented hyperosmotic avoidance. However, the behavioral phenotype could not be restored to the WT by the reconstitution of *flp-21* genomic DNA driven by its promoter of 4.1 kb *flp-21p* (Macosko et al., 2009) and *ver-2p* of 2.7 kb (Figure 2D). The results suggest that FLP-4 may mediate ADL excitation of ASH.

NPR-4 and NPR-5 are known FLP-4 receptors (Cohen et al., 2009; Frooninckx et al., 2012). The gene *npr-5* displays trace expression in ASH neurons by single cell RNA sequencing.^2^ We thus used *npr-4* and *npr-5* mutant animals to test animals’ hyperosmotic avoidance. Loss-of-function (lof) mutant animals of *npr-4*(*tm1782*) and *npr-5*(*ok1583*) showed significantly decreased hyperosmotic avoidance compared to WT N2 (Figure 2E; Supplementary Figure S2B). *npr-4* and *npr-5* rescue expression in its expression cells driven by its promoters *npr-4p* and *npr-5p* were able to restore WT hyperosmotic avoidance, respectively (Figure 2E; Supplementary Figure S2B). However, only *npr-5* genetic rescue in ASH restored the WT hyperosmotic avoidance. Furthermore, animals of the *flp-4*(*sy1606*), *npr-5*(*ok1583*), and double knockout (*DKo*) of *flp-4*(*sy1606*); *npr-5*(*ok1583*), displayed almost similar defects in hyperosmotic avoidance. Only double gene rescued animal of ADL::*flp-4*; ASH::*npr-5*; *DKo* restored WT behavior; both single gene rescue animals of ADL::*flp-4*; *DKo* and ASH::*npr-5*; *DKo* phenotypically copied animals of single *flp-4* and *npr-5* mutant and the double mutant (Figure 2E). The results suggest that FLP-4 and NPR-5 function in the same signal pathway to regulate hyperosmotic avoidance.

As FLP-4/NPR-5 signaling between ADL and ASH mediates hyperosmotic avoidance upregulation, it should act to augment the ASH Ca^2+^ responses to hyperosmolality. As expected, *flp-4*(*sy1606*) and *npr-5*(*ok1583*) animals showed similar decreased ASH Ca^2+^ responses to the stimulation of 0.88 Osm glycerol/M13 solution. In contrast, ADL::*flp-4*; *flp-4* and ASH::*npr-5*; *npr-5* rescue animals showed WT Ca^2+^ responses (Figures 2F,G; Supplementary Figure S2C). In addition, chemogenic inhibition of ADL significantly reduced ASH Ca^2+^ responses to medium hyperosmolality of 0.88 Osm (Figures 2H,I; Supplementary Figure S2D).

The above results support that ADL positively regulates *C. elegans*’ avoidance of medium hyperosmolality and augments ASH sensory response *via* the FLP-4/NPR-5 signaling pathway.

### RIM suppresses avoidance and receives sensory input from ASH *via* the glutamate signaling pathway

**Figure 3:**
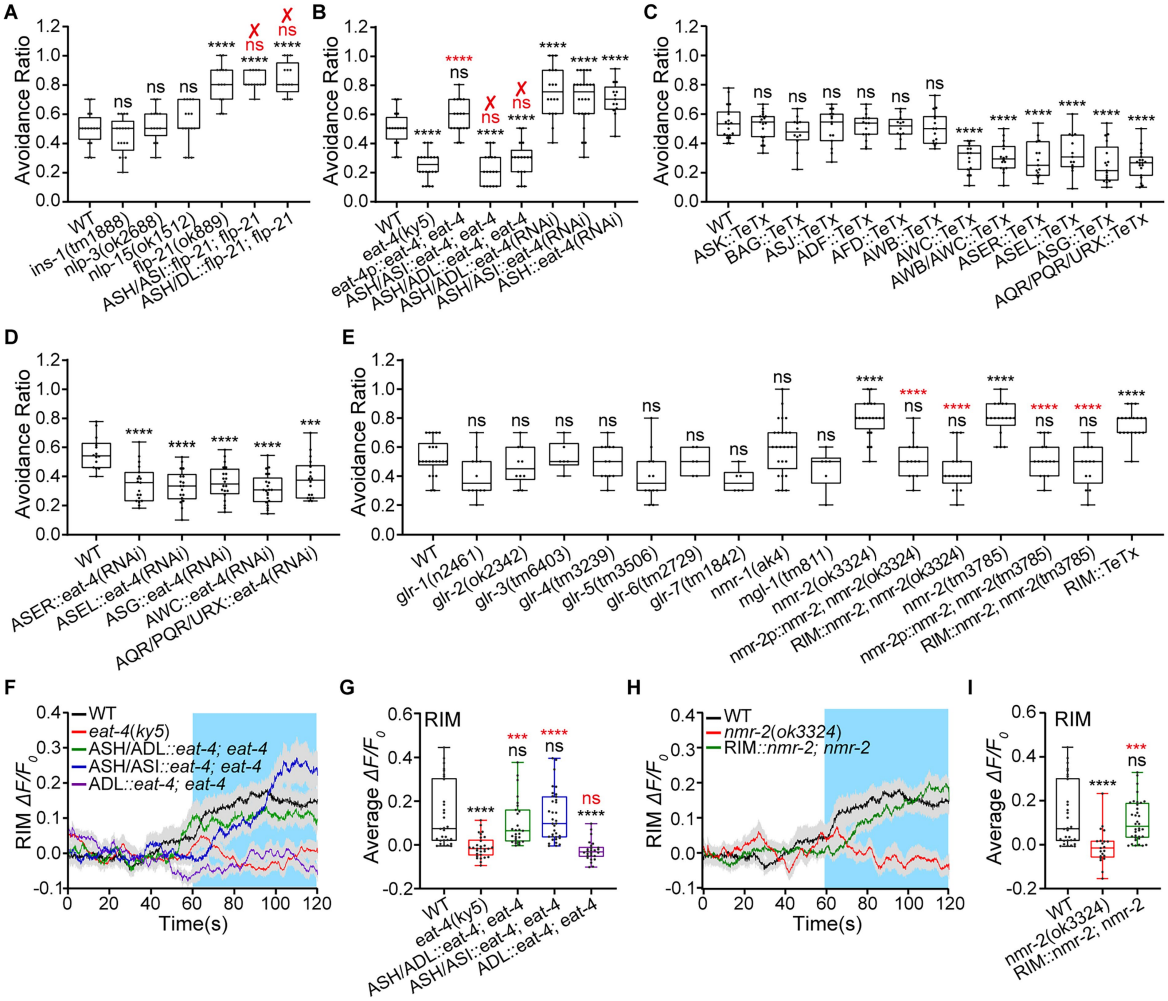
RIM suppresses animal hyperosmotic avoidance, and ASH inhibits RIM activities *via* the glutamate/NMR-2 signaling pathway. **(A–E)** Ratio of avoidance of a droplet of the 0.88 Osm glycerol/M13 hyperosmotic solution in animals of indicated genotypes. “✘” indicates a failure to restore WT behavior and the genetic rescue. **(F–I)** The somal calcium transients in RIM interneurons in response to the hyperosmolality in the WT N2, mutant, and transgenic animals of indicated genotypes. **(F,H)** Curves of Ca^2+^ transients presented as means (solid traces) ± SEM (gray shading) with cerulean background indicating the application of 0.88 Osm glycerol/M13 solution; G and I, box plots of the average intensity of Ca^2+^ signals during stimulation with each dot representing the data from each tested worm. Heat maps of Ca^2+^ signals are shown in Supplementary Figure S3. Statistical significance is indicated as and in different colors: ns, not significant, ^***^p < 0.001, and ^****^p < 0.0001 in different colors; black, the tested vs. the WT; red, the gene rescued vs. the related mutant.

Our results indicate that ASH inhibits ADL Ca^2+^ responses to medium hyperosmolality of 0.88 Osm glycerol/M13, and that there is an interaction between ASH and ADL nociceptive neurons. ADL upregulated ASH sensory response to the medium hyperosmolality by FLP-4 signaling. ASH inhibits ADL hyperosmotic sensory response. However, the underlying mechanism still needs study. ASH is a glutamatergic and neuropeptidergic neuron. It is known to release glutamate and several neuropeptides, FLP-21, INS (INSulin related)-1, NLP-3, and NLP-15, for neurotransmission (see footnote 1; Li and Kim, 2008; Serrano-Saiz et al., 2013). Thus, we identified the neurotransmitter/s, neuropeptide/s or/and glutamate, by which ASH generates avoidance of hyperosmotic shock and downregulates ADL activity. We first used mutant animals of neuropeptides released by ASH and EAT-4 to assay hyperosmotic avoidance behavior. EAT-4 is a vesicular L-glutamate transporter essential for filling glutamate into synaptic vesicles and, thus, glutamatergic neurotransmission (Lee et al., 1999; Bellocchio et al., 2000). Among mutant animals of *ins-1*, *nlp-3*, *nlp-15*, and *flp-21*, only the *flp-21*(*ok889*) displayed augmented hyperosmotic avoidance that contradicts the expected phenotype. In addition, the behavioral phenotype was not restored to the WT in transgenic animals expressing *flp-21* in ASH/ADL and ASH/ASI (Figure 3A). Thus, ASH is not likely to play its role by neuropeptidergic signaling. It may act *via* glutamatergic signaling. As expected, our data showed that the *eat-4*(*ky5*) animals were less sensitive to medium hyperosmolality of 0.88 Osm. Although *eat-4* rescue expression in its expression cells driven by its promoter *eat-4p* (5.6 kb) restored WT hyperosmotic avoidance in the transgenic animal, *eat-4* re-expression in ASH/ASI and ASH/ADL showed no effect on the behavior (Figure 3B). *eat-4* is widely expressed in the *C. elegans* nervous system, including sensory neurons, interneurons, and motor neurons.^3^ It sounds reasonable that *eat-4* reconstitution in ASH alone cannot restore the WT hyperosmotic avoidance behavior. We thus used ASH-specific RNAi knockdown of *eat-4* by cell-selective promoters, *srv-11p, gpa-11p,* and *sra-6p*, to identify the role of glutamate signaling in hyperosmotic avoidance. As expected, the genetically knocked-down animals of ASH (driven by *srv-11p* of 1.9 kb)::*eat-4*(RNAi), ASH/ADL::*eat-4*(RNAi), and ASH/ASI::*eat-4*(RNAi) showed a significantly increased avoidance ratio (Figure 3B). All three transgenic animals displayed similar behavioral phenotypes, supporting ASH acting in hyperosmotic avoidance through glutamate signaling.

The inconsistency of behavioral phenotype in animals of *eat-4* genetic rescue and knockdown in ASH suggests that glutamatergic sensory neurons besides ASH and ADL may be involved in hyperosmotic avoidance. We then used TeTx neurotransmission inhibition to screen glutamatergic sensory neurons preliminarily. TeTx neurotransmission inhibition or elimination in AWC, ASE, ASG, AQR, and PQR, but not in ASK, BAG (BAG-like Dendritic Ending), ASJ (Amphid Single Cilium J), ADF (Amphid Dual Ciliated Ending F), AFD (Amphid Finger-like Endings D), and AWB (Amphid Wing Neuron B), reduced hyperosmotic avoidance (Figure 3C). We further used neuron-specific knockdown of *eat-4* to confirm the result of genetic neurotransmission inhibition. As shown in Figure 3D, the *eat-4* knockdown in the candidate sensory neurons identified by *TeTx* manipulation diminished hyperosmotic avoidance. The results indicate that in addition to ASH and ADL, multiple sensory neurons are involved in hyperosmotic sensation and avoidance behavior.

We focused the present study on the mechanism of interaction between ADL and ASH and the function of this interaction. We next identified the glutamatergic receptor/s involved in ASH regulation of hyperosmotic avoidance. Among the mutant animals we tested, *nmr-2*(*ok3324*) and *nmr-2*(*tm3785*) displayed notably augmented hyperosmotic avoidance. The reconstitution of *nmr-2* in its expression cells driven by its promoter *nmr-2p* (4.9 kb) restored the WT behavioral phenotype (Figure 3E). NMR-2 is an NMDA glutamate receptor (Kano et al., 2008). It is expressed in sensory neurons, motor neurons, and interneurons RIM (see footnote 3). ASH may use the same neurotransmitter for functioning in animal hyperosmotic avoidance and negative regulation of ADL. ASH is postsynaptic but not presynaptic to ADL. ADL expresses a tyramine receptor TYRA-3, an octopamine receptor SER (SERotonin/octopamine receptor)-6, but no glutamate receptor (see footnotes 1, 3). Thus, ASH may inhibit ADL activity through the relay by intermediate neuron/s. RIM expresses glutamatergic NMR-2 and releases tyramine and glutamate (see footnote 1). Therefore, we focused on testing RIM. We used *nmr-2* genetic rescue and RIM-specific TeTx neurotransmission inhibition to test the RIM function in hyperosmotic avoidance and ADL activity. As expected, the RIM-specific (driven by *gcy-13p* of 2.3 kb) *nmr-2* rescue and *TeTx* expression restored the WT behavior and displayed augmented hyperosmotic avoidance, respectively (Figure 3E). We further employed RIM::chemogenetic inhibition to examine RIM function in adult animal behavior. Specific RIM inhibition by expressing HisCl1 and applying 10 mM histamine made the animal more sensitive to hyperosmolality by 0.88 Osm glycerol/M13 than the WT (Supplementary Figure S3A). These results support that the NMR-2 signaling in RIM interneurons mediates ASH function in hyperosmotic avoidance and possibly downregulation of ADL activity.

We next examined RIM somal Ca^2+^ responses to 0.88 Osm glycerol/M13 solution in *eat-4* and *nmr-2* mutant and genetically rescued animals to confirm glutamate/NMR-2 signaling for neurotransmission from ASH to RIM. The RIM Ca^2+^ responses to 0.88 Osm osmolality in the *eat-4*(*ky5*) animal significantly decreased. The signals were fully restored to the WT by the extrachromosomal re-expression of *eat-4* cDNA in ASH, using two cell-specific promoters, *gpa-11p* (in ASH and ADL) and *sra-6p* (in ASH and ASI) with an expression overlap is ASH. However, reconstitution of *eat-4* in ADL, which is postsynaptic to RIM, did not rescue the RIM Ca^2+^ responses to 0.88 Osm osmolality (Figures 3F,G; Supplementary Figure S3B). In addition, the 0.88 Osm hyperosmolality-evoked RIM Ca^2+^ responses in the *nmr-2*(*ok3324*) significantly decreased and restored to the WT in the RIM-specific *nmr-2* rescued animal (Figures 3H,I; Supplementary Figure S3C). There is an inconsistency between behavioral assay (Figure 3B) and calcium imaging (Figures 3F,G) results in animals of *eat-4* rescue in ASH/ADL or ASH/ASI. This inconsistency is expected. Because multiple glutamatergic neurons are required for normal hyperosmotic avoidance, and ASH activates its postsynaptic RIM neurons by chemical synapse connection, the process does not need other glutamatergic neurons. These data support that RIM relays ASH function in animal hyperosmotic avoidance and possibly ASH down-regulation of ADL.

### Rim inhibits ADL by tyramine/TYRA-3 signaling

**Figure 4:**
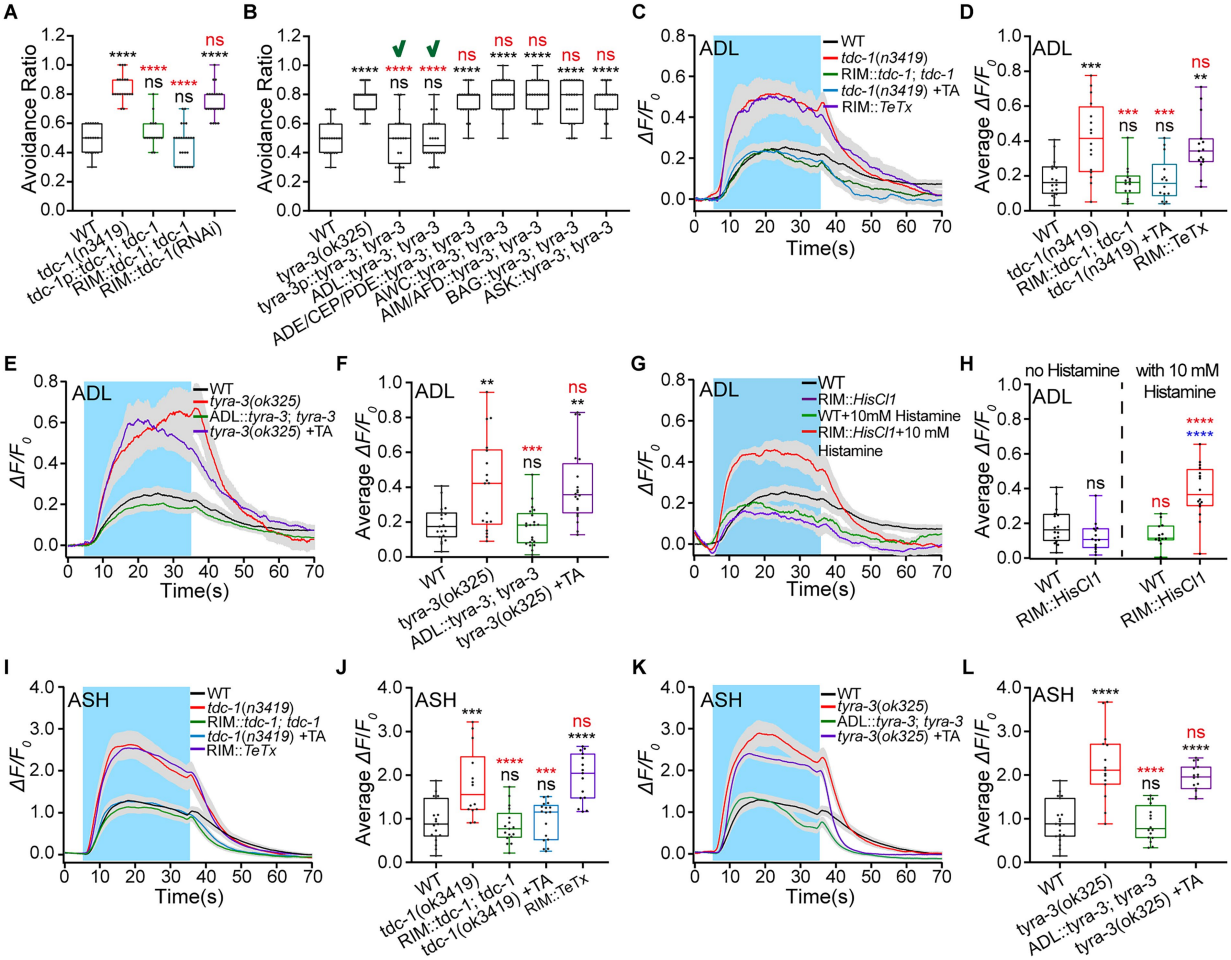
RIM inhibits hyperosmotic avoidance by inhibiting ADL *via* tyramine/TYRA-3 signaling. **(A,B)** Ratio of avoidance of a droplet of the 0.88 Osm glycerol/M13 hyperosmotic solution in animals of indicated genotypes. “**√**” indicates the success of restoring WT behavior by the genetic rescue indicated. **(C–L)** The somal calcium signals in paired ADL **(C–H)** and ASH **(I–L)** sensory neurons in response to the application of hyperosmotic solution in WT, mutant, and transgenic animals of indicated genotypes. **(C,E,G,I,K)** Curves of Ca^2+^ transients presented as means (solid traces) ± SEM (gray shading) with cerulean background indicating the application of 0.88 Osm glycerol/M13 solution; **(D,F,H,J,L)** Box plots of the average intensity of Ca^2+^ signals of the ON response during glycerol/M13 solution perfusion with each dot representing the data from each tested worm. Heat maps of Ca^2+^ signals are shown in Supplementary Figure S4. Statistical significance is indicated as and in different colors: ns, not significant, ^**^p < 0.01, ^***^p < 0.001, and ^****^p < 0.0001; black, the tested vs. the WT; red, the gene rescued vs. the related mutant, or the histamine treated vs. histamine untreated of the same genotype (in **H**); blue, the tested vs. the WT under histamine treatment (in **H**).

The above results suggest that RIM interneurons relay ASH inhibition to ADL. RIM is tyraminergic, and ADL expresses the tyraminergic receptor TYRA-3 (see footnotes 1, 3). Thus, RIM modulates ADL activities, possibly through tyramine/TYRA-3 signaling. Tyramine is a monoamine neuromodulator (Alkema et al., 2005). It is synthesized from tyrosine catalyzed by tyrosine decarboxylase TDC-1. We thus used *tdc-1* mutant and RIM-specific knocked-down animals to examine the functions of tyraminergic signaling in hyperosmotic avoidance and ADL Ca^2+^ responses to hyperosmotic stimulation. The *tdc-1*(*n3419*) animal displayed significantly augmented avoidance of 0.88 Osm glycerol/M13 solution. This behavioral phenotype was fully restored to the WT by *tdc-1* genetic rescue expression in its expression neurons (RIM and RIC) and RIM alone, driven by *tdc-1* promoter (3.0 kb) and *gcy-13p* (2.3 kb), and by administration of exogenous tyramine (5 mM). Furthermore, the RIM-specific *tdc-1* knockdown showed the same behavioral effect as the *tdc-1* mutation (Figure 4A; Supplementary Figure S4A). This result supports that RIM modulates hyperosmotic avoidance by releasing tyramine. However, the postsynaptic tyraminergic receptor/s need/s to be identified. *Caenorhabditis elegans* expresses tyraminergic receptors SER-2, TYRA-2, TYRA-3, and LGC (Ligand-Gated ion Channel)-55. We thus used the receptor mutant animals to test which receptor/s function/s in animal hyperosmotic avoidance. Among *ser-2*, *tyra-2*, *tyra-3*, and *lgc-55* mutant animals, only the *tyra-3*(*ok325*) showed non-WT behavior, a significantly augmented hyperosmotic avoidance (Supplementary Figure S4B). The hyper-avoidance was restored to the WT by *tyra-3* rescue expression in its expression cells and ADL alone, but not in ASK, AWC, and BAG neurons, or neuronal sets ADE (Anterior DEirid Neuron)/CEP (CEPhalic Sensory Neuron)/PDE (Posterior DEirid) and AIM (Anterior Interneuron M)/AFD (Amphid Dual Ciliated Ending F; Figure 4B). These results indicate that RIM regulates *C. elegans* hyperosmotic avoidance by neurotransmission to ADL through tyramine/TYRA-3 signaling.

RIM may use the same signaling pathway to regulate ADL activities as it does to modulate hyperosmotic avoidance. We first examined the tyramine role in ADL Ca^2+^ responses to 0.88 Osm glycerol/M13. ADL Ca^2+^ signals in *tdc-1*(*n3419*) and RIM::*TeTx* neurotransmission-eliminated animals were augmented similarly. The increased Ca^2+^ response in the mutant animal was restored to the WT by the RIM-specific *tdc-1* genetic rescue and the treatment with 50 μM exogenous tyramine (Figures 4C,D; Supplementary Figure S4C). This suggests that RIM inhibits ADL through tyramine signaling. Logically, ADL Ca^2+^ responses to 0.88 Osm glycerol/M13 should change similarly in *tyra-3*(*ok325*) and *tdc-1*(*n3419*) animals. As expected, the ADL Ca^2+^ signals in the *tyra-3*(*ok325*) significantly increased and were restored to the WT by ADL::*tyra-3* genetic rescue but not by the application of exogenous tyramine (Figures 4E,F; Supplementary Figure S4D). In addition, RIM-specific chemogenetic inhibition increased the ADL Ca^2+^ signals (Figures 4G,H; Supplementary Figure S4E). These results indicate that RIM interneurons inhibit ADL sensory neurons by tyramine/TYRA-3 signaling.

We demonstrated that ASH excites RIM by glutamate/NMR-2 signaling, RIM inhibits ADL *via* the neurohumoral tyramine/TYRA-3 pathway, and ADL excites ASH through FLP-4/NPR-5 signaling. Three types of neurons form a negative feedback circuit. Then, RIM should decrease Ca^2+^ responses to hyperosmolality in ASH as in ADL. We thus employed an experimental strategy like the one we used for examining the RIM effect on ADL to test the RIM regulation of ASH. Indeed, the changes in ASH Ca^2+^ responses to 0.88 Osm glycerol/M13 in *tdc-1* and *tyra-3* mutant, the genetically rescued, and tyramine-treated animals, almost entirely copied the changes in ADL Ca^2+^ signals (Figures 4I–L; Supplementary Figures S4F,G). These results support that ASH, RIM, and ADL form a negative feedback circuit. In this circuit, ASH excites RIM; RIM inhibits ADL, and ADL excites ASH. Thus, ASH exciting RIM removes or reduces ADL exciting ASH. This neuronal signal integration modality is another illustration of disexcitation reported in our previous study (Liu et al., 2019).

In the natural habitat of *C. elegans*, the osmolality changes are usually gradual instead of acute like in the drop test. Furthermore, environmental hyperosmolality may increase nematode internal osmolality. *Caenorhabditis elegans* senses osmotic upshifts *via* signaling that requires the cGMP-gated sensory channel subunit TAX-2 in body cavity sensory neuron URX (Unknown Receptor, not Ciliated X) and that generates increased aversion behavior (Yu et al., 2017). We performed a similar droplet assay used by this previous study to test whether the ASH/RIM/ADL circuit function in the behavioral response of internal osmolality upshifts (Yu et al., 2017). The WT N2 animal displayed a rapid increase of aversion behavior in 1 min in response to hyperosmolality (in Osm) of 0.41, 0.49, 0.62, 0.74, and 0.88 by glycerol/M13 solutions and was quickly paralyzed by immersion in solutions of higher hyperosmolality of 0.74 Osm and 0.88 Osm (Supplementary Figures S5A,B). We further used transgenic animals of ASH::*TeTx*, ADL::*TeTx*, and RIM::*TeTx* to test hyperosmotic behavioral response to the treatment of 0.62 Osm glycerol/M13. Our data showed that all transgenic animals responded to the hyperosmolality treatment similarly to the WT N2 (Supplementary Figures S5C,D). This result does not support that ASH/RIM/ADL circuit functions in aversive behavior responding to the upshift of internal osmolality.

### ASH/RIC/AIY feedforward circuit enhances hyperosmotic avoidance

So far, our results indicate that ASH down-regulates ADL sensory responses to and *C. elegans* avoidance of medium osmolality through the ASH/RIM/ADL negative feedback circuit. Since ASH is the main osmolality-sensitive sensory neuron, it should generate and regulate hyperosmotic avoidance *via* other neuronal circuits. ASH synaptically connects with forward and backward command interneurons, such as AVA (Anterior Ventral Process A), AVB (Anterior Ventral Process B), AVD (Anterior Ventral Process D), and AVE (Anterior Ventral Process E). ASH connects with interneurons controlling or modulating locomotion by chemical and electric synapses (White et al., 1986; Cook et al., 2019).^4^ Among the interneurons postsynaptic to ASH, RIC regulates the avoidance of heavy metal ions Cu^2+^ (Guo et al., 2015, 2018) and aversive odor 2-nonanone (Kimura et al., 2010) by neurohumoral octopamine. ASH may regulate hyperosmotic avoidance through interneuron RIC. We thus evaluated the RIC function in hyperosmotic avoidance.

**Figure 5:**
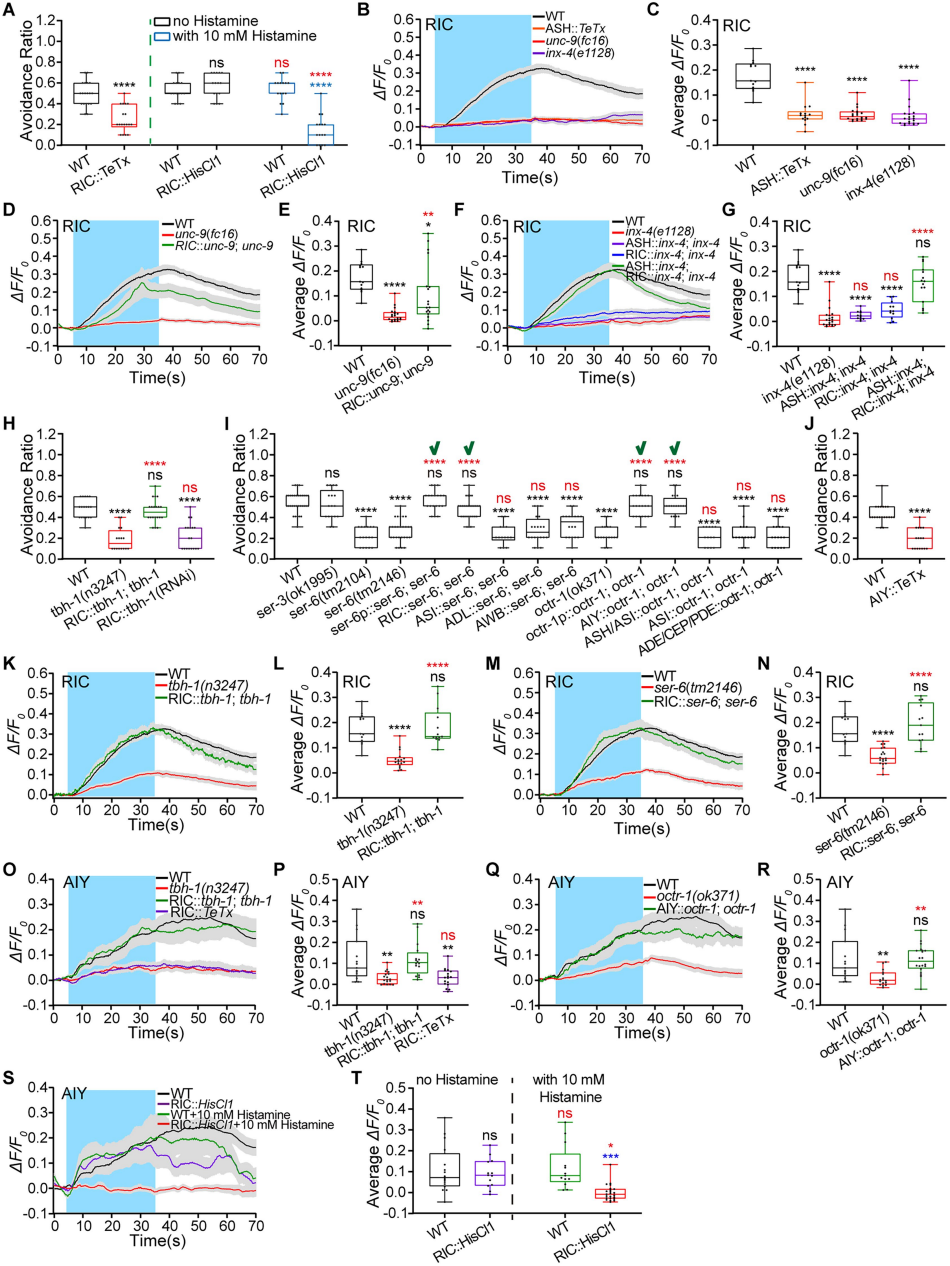
Signaling pathways of Octopamine/SER-6 in RIC and octopamine/OCTR-1 in AIY promote animals’ hyperosmotic avoidance. **(A)** Ratio of avoidance of the hyperosmolality of 0.88 Osm by glycerol/M13 in wild-type (WT) N2 as control and transgenic animals of indicated genotypes. **(B–G)** The somal calcium signals in RIC interneurons in response to the hyperosmolality of 0.88 Osm in WT, mutant, and transgenic animals of indicated genotypes. **(B,D,F)** Curves of Ca^2+^ transients presented as means (solid traces) ± SEM (gray shading) with cerulean background indicating the application of 0.88 Osm glycerol/M13 solution; **(C,E,G)** box plots of the average intensity of Ca^2+^ signals during stimulation with each dot representing the data from each tested animal or test. **(H–J)** Ratio of avoidance of the droplet of the hyperosmotic solution in WT N2, mutant, and transgenic animals of indicated genotypes. “**√**” indicates the success of restoring WT behavior by the genetic rescue indicated. **(K–T)** The somal calcium signals in RIC **(K–N)** and AIY **(O–T)** interneurons in response to the hyperosmolality of 0.88 Osm in WT, mutant, and transgenic animals of indicated genotypes. **(K,M,O,Q,S)** Curves of Ca^2+^ transients presented as means (solid traces) ± SEM (gray shading) with cerulean background indicating the application of 0.88 Osm glycerol/M13 solution; **(L,N,P,R,T)** box plots of the average intensity of Ca^2+^ signals during stimulation with each dot representing the data from each individual tested worm. Heat maps of Ca^2+^ signals are shown in Supplementary Figures S6, S7. Statistical significance is indicated as and in different colors: ns, not significant, ^*^p < 0.05, ^**^p < 0.01, ^***^p < 0.001, and ^****^p < 0.0001 in different colors; black, the tested vs. the WT; red, the gene rescued vs. the related mutant, or the histamine treated vs. histamine untreated of the same genotype (in **A,T**); blue, the tested vs. the WT under histamine treatment (in **A,T**).

We first eliminated RIC neurotransmission and inhibited its activity by RIC::*TeTx* and RIC::chemogenetic inhibition to test RIC’s role in hyperosmotic avoidance. These two types of neuronal manipulations similarly reduced animal hyperosmotic avoidance, supporting to the RIC enhancement effect on the behavior (Figure 5A). RIC should display a response to hyperosmolality, possibly by excitation from ASH through electrical synapses. We next used Ca^2+^ imaging to assay the RIC response. As expected, RIC displayed robust Ca^2+^ responses to hyperosmolality of 0.88 Osm in the WT N2 animal (Figures 5B,C; Supplementary Figure S6A). ASH and RIC express the innexin family of gap junction proteins INX-4 (in both neurons) and UNC-9 (in RIC; Altun et al., 2015; see footnote 4). A strong TeTx expression in ASH disrupts ASH’s gap junctions and, thus, RIC excitation in response to noxious Cu^2+^ stimulation (Guo et al., 2015). If the RIC hyperosmotic response resources directly from ASH by gap junctions, it should majorly decrease or even disappear in the mutant animals of these two innexin proteins and ASH::TeTx transgenic worm. As expected, the RIC Ca^2+^ responses to the medium hyperosmolality almost disappeared in *unc-9*(*fc16*), *inx-4*(*e1128*), and ASH::*TeTx*, and were restored to the WT in transgenic animals of the RIC::*unc-9*;*unc-9* and ASH::*inx-4*; RIC::*inx-4*; *inx-4* (Figures 5B–G; Supplementary Figures S6A–C). All these results support that ASH excites RIC *via* gap junction.

RIC is octopaminergic. It may modulate hyperosmotic avoidance through octopaminergic signaling. Octopamine is biosynthesized from tyramine catalyzed by tyramine β-hydroxylase TBH-1 (Chase and Koelle, 2007). We then used *tbh-1* mutant and rescued animals to test the octopamine role in hyperosmotic avoidance. The *tbh-1*(*n3247*) mutant displayed reduced hyperosmotic avoidance; the RIC-specific *tbh-1* rescued and exogenous octopamine-treated *tbh-1*(*n3247*) animals showed the WT behavioral phenotype (Figure 5H; Supplementary Figure S6D). Moreover, the RIC-specific *tbh-1* knocked-down animal phenocopied the *tbh-1* mutant (Figure 5H). *Caenorhabditis elegans* expresses octopaminergic receptors OCTR-1 (OCTopamine Receptor 1), SER (SERotonin/octopamine receptor)-3, and SER-6. We next employed receptor mutant animals to identify the octopamine receptor/s involved in hyperosmotic avoidance. *Ser-6* and *octr-1* mutant animals displayed significantly reduced hyperosmotic avoidance. The behavioral defects were eliminated by the gene rescue driven by their promoters (Figure 5I). SER-6 and OCTR-1 are expressed in neurons including RIC and AIY, respectively (see footnote 4). Octopamine from RIC may regulate hyperosmotic avoidance by two signaling pathways: autocrine SER-6 and OCTR-1 in AIY. As expected, the genetic reconstitution of *ser-6* in RIC and *octr-1* in AIY but no other *ser-6*-expressing or *octr-1*-expressing neurons restored the WT behavioral phenotype in transgenic animals (Figure 5I). In addition, AIY::*TeTx* and AIY::chemogenetic inhibition similarly suppressed animals’ hyperosmotic avoidance (Figure 5J; Supplementary Figure S6E). These behavioral test results support the hypothesis of two signaling pathways.

We next monitored RIC and AIY Ca^2+^ responses to the medium hyperosmolality to support the behavioral conclusion. Without exception, all Ca^2+^ image data were as expected. The hyperosmolality-elicited somal calcium transients of RIC significantly decreased in the *tbh-1*(*n3247*) animal and restored to the WT in the RIC-specific gene rescued animal (Figures 5K,L; Supplementary Figure S6F). In the same way, the RIC Ca^2+^ signals changed in the *ser-6*(*tm2146*) and RIC-specific *ser-6* rescued animals (Figures 5M,N; Supplementary Figure S6G). The AIY Ca^2+^ responses to the hyperosmolality were nearly eliminated in *tbh-1*(*n3247*) and RIC::*TeTx* animals and restored to the WT in the RIC-specifically *tbh-1* rescued animal (Figures 5O,P; Supplementary Figure S7A). AIY Ca^2+^ responses in *octr-1* mutant and AIY rescued animals changed similarly to the RIC signals in *ser-6* mutant and RIC-rescued animals (Figures 5Q,R; Supplementary Figure S7B). In addition, RIC chemogenetic inhibition reduced the AIY Ca^2+^ signals (Figures 5S,T; Supplementary Figure S7C).

All the above results support that ASH, RIC, and AIY form a feedforward circuit to upregulate hyperosmotic avoidance. The signaling pathways in this circuit are direct signal flow from ASH to RIC through electric synapses, autocrine octopamine/SER-6 signaling in RIC, and octopamine/OCTR-1 pathway between RIC and AIY.

## Discussion

**Figure 6:**
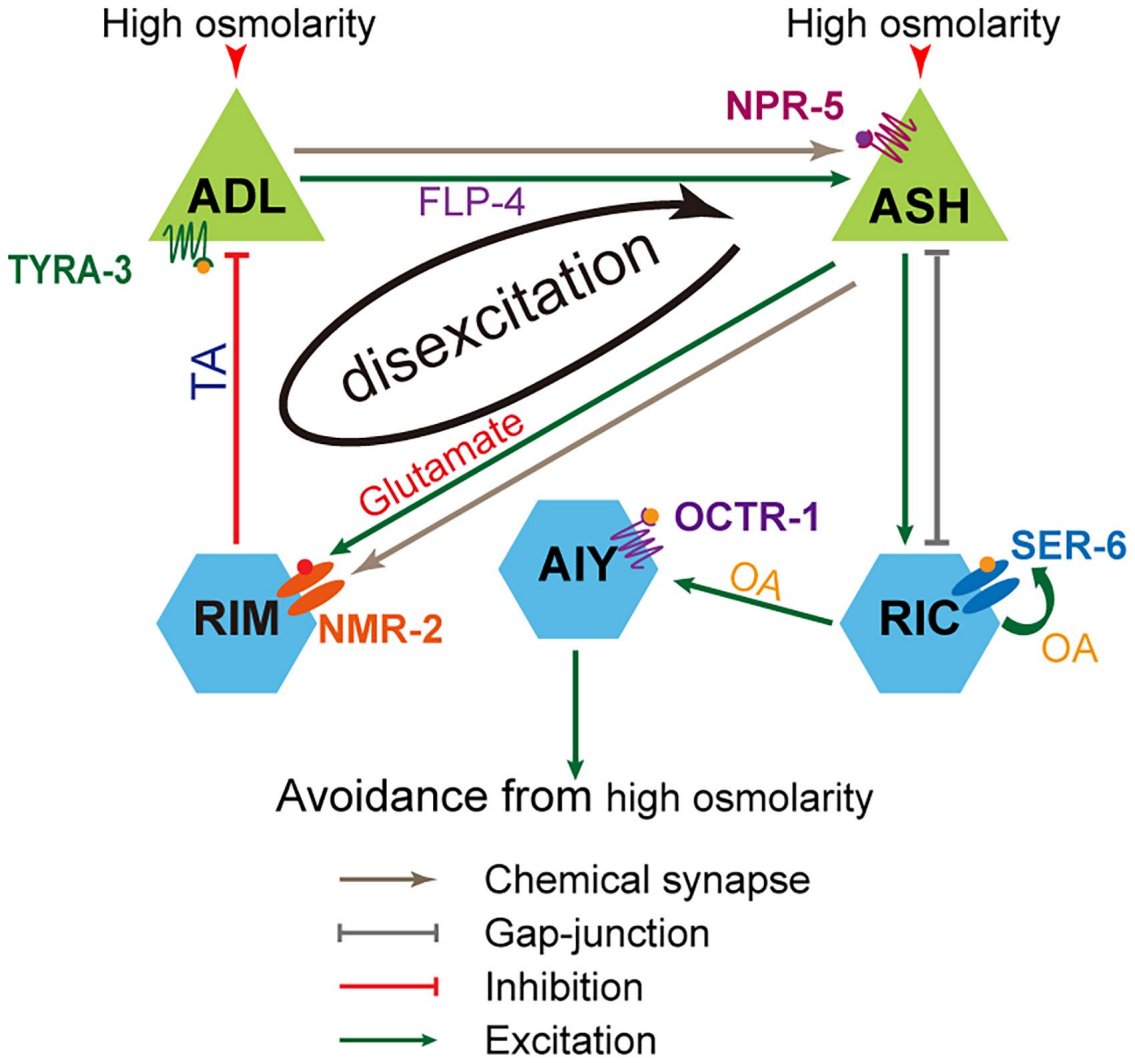
Working model for neuronal circuitry mechanism underlying the regulation of C. *elegans* avoidance of the mild and medium hyperosmolality. ASH and ADL interact and form a negative feedback circuit with the involvement of RIM interneurons. In the circuit, ADL promotes ASH activities *via* the FLP-4/NPR-5 signaling, ASH excites RIM by glutamate/NMR-2 signaling, and RIM suppresses ADL *via* tyramine/TYRA-3 signaling. Thus, ASH exciting RIM reduces or removes ADL exciting ASH. The neuronal information integration modality in the circuit is disexcitation. Besides, ASH elicits hyperosmotic avoidance through ASH/RIC/AIY feedforward circuit. In this circuit, octopamine/SER-6 signaling acts as an auto-excitation.

Sensory neurons ASH and ADL are primary and secondary nociceptors of noxious stimuli. Both neurons are necessary for *C. elegans* to fine-tune hyperosmotic sensation and proper avoidance behavior. Two types of neurons dynamically interact *via* the negative feedback circuit consisting of ASH, RIM, and ADL. Upon hyperosmolality stimulation, ASH is excited, its excitation transmits directly to interneurons RIM *via* chemical synapses, RIM inhibits hyperosmolality-sensitive ADL *via* tyramine/TYRA-3, and ADL enhances ASH activity by FLP-4/NPR-5 signaling pathway. The modality of neuronal signal integration in this circuit is disexcitation, which was newly identified in our previous study (Liu et al., 2019). The activities of neurons in the circuit are dynamic and osmolality dependent. Mild and medium osmotic pressures (<1.4 Osm) cause lower excitatory levels in ASH and less intense ASH inhibition on ADL, thus, on ADL’s ability to enhance ASH activity. In contrast, high osmotic pressures (>1.4 Osm) elicit higher excitatory levels in ASH, more intense ASH inhibition on ADL, and thus deterioration or even elimination of ADL exciting ASH, that is, disexcitation of ASH. These dynamic neuronal activities and reciprocal interaction between sensory neurons endow animals with higher sensitivity levels of hyperosmolality below medium pressure. Besides, ASH upregulates hyperosmotic avoidance through the ASH/RIC/AIY feedforward circuit. Interestingly, neuroendocrinal RIC releases octopamine to enhance AIY activity through OCTR-1 signaling and augment the activity of itself (auto-excite) by acting on the SER-6 receptor. The molecular and circuital mechanisms are illustrated in Figure 6. In addition to ASH and ADL, multiple sensory neurons, including ASH, ADL, ASE, ASG, AWC, AQR, and PQR, are involved in moderate hyperosmotic avoidance.

The hyperosmolality-sensitive ADL differently functions in hyperosmotic avoidance of the same stimulation of 2 M glycerol in different nematode species, *C. elegans* and *Pristionchus pacificus* (Srinivasan et al., 2008), and intensity-dependently acts in *C. elegans* avoidance of moderate hyperosmolality (0.41 and 0.88 Osm) and high hyperosmolality (1.37 and 2.29 Osm, this study). The differentiated roles of intensity-or concentration-dependent sensations in animal behaviors, which display widely in the animal species, are intrinsic traits essential for animal adaptation to environmental changes. In addition to the ADL case, there are the following instances. The concentrations-dependent sensations of isoamyl alcohol mediated by ODR-3 in AWC responding to only lower concentrations of the odorant and ASH responding to only higher concentrations of odorant induce odor attraction and avoidance behaviors (Hilliard et al., 2005; Bargmann, 2006; Yoshida et al., 2012; Duan et al., 2020). The sensation of low salt concentration mediated by sodium-selective epithelial sodium channels (ENaC) in mice (Chandrashekar et al., 2010) and *Drosophila* (Liu et al., 2003) directs attractive behavior. In contrast, the high concentration salt taste transduced by TRPM5 (Transient Receptor Potential Melastatin) channel elicits aversive behavioral responses in mice (Oka et al., 2013), while PPK11 (pickpocket) and PPK19 (ENaC members) in *Drosophila* (Liu et al., 2003) are possibly involved in high salt concentration avoidance. The ASH sensory information of low and high concentrations of quinine, decoded in AIB interneurons by GLR-1 (GLutamate Receptor) and GLR-5 receptors, is involved in the reversal initiation and feeding suppression, respectively (Zou et al., 2018). The sense of low concentration of diacetyl with ODR-10 (odorant response abnormal protein 10) as a receptor in AWA sensory neurons directs the attractive response, in contrast, that of the high odor concentration by SRI (Serpentine Receptor, class I)-14 receptor (a G Protein-Coupled Receptor) in ASH mediates the odor avoidance (Taniguchi et al., 2014). The sensations of low and high concentrations of the bacteria-derived volatile chemical dimethyl trisulfide (DMTS), depending on SRI-14 in AWC and ASH, mediate attractive and aversive behavioral responses, respectively. In this behavior, AWC detects a wide range of low to high DMTS concentrations, and ASH detects a narrow range of high concentrations (Choi et al., 2022).

Physiological regulations and behaviors depend more on neuronal circuits than on individual neurons. Neural signal integration is the basis of neurocircuit functions. The modalities of neuronal information integration are evolutionarily conserved in animals, including humans (Reigl et al., 2004; Sporns and Kotter, 2004; Liu et al., 2019). The neural information analysis and integration in neuronal circuits are based on excitatory and inhibitory neural signal transmissions between the presynaptic and postsynaptic neurons. The combination of two types of neurotransmissions results in a few modalities or models of neural information integration in the neural circuits. The excitatory and inhibitory modalities are common in animals. The known models of neural information integration function as follows. A central disinhibition or unmasking process in the cerebral cortex functions in illusory pain in humans (Craig and Bushnell, 1994; Craig et al., 1996). This modality also acts in the mammalian basal ganglia to facilitate the initiation of motor programs (White and Hall, 2012). A disinhibitory circuit cooperates with a stimulatory circuit to promote the initiation of reversals in *C. elegans* (Piggott et al., 2011). The disinhibition in the ADF/RIC/SIA feedforward circuit functions to augment pumping under food supply conditions (Liu et al., 2019). The gate control theory of pain proposed that the relative balance of activity in nociceptive and nonnociceptive afferents controls the transmission and perception of pain. By engaging inhibitory interneurons in the dorsal horn, the activation of nonnociceptive sensory neurons closes a “gate” for afferent transmission of nociceptive signals that can be opened by the activation of nociceptive sensory neurons (Melzack and Wall, 1965). Such interactions can also occur at many supraspinal relay centers (Basbaum, 2021). Reciprocal inhibition engages widely in regulating or controlling movement (Brown, 1914; Crone et al., 1987; Pearson, 1993; Friesen, 1994; Marder et al., 2005; Drew and Kiehn, 2021), nociception (Guo et al., 2015), decision-making (Li et al., 2012), and fast escape (Liao and Fetcho, 2008; Satou et al., 2009). The disexcitation found in ADF/RIC/AWB/ADF circuit generates the homeostasis of pharyngeal pumping and 5-HT production in food-sensing ADFs under food supply and deprivation conditions (Liu et al., 2019). This modality in ASH/RIM/ADL negative feedback circuit (this study) osmolality-dependently regulates hyperosmotic sensation and avoidance behavior, in particular, augments the hyperosmotic sense and avoidance of mild and medium hyperosmolality.

In animals, including *C. elegans*, neuropeptide genes are expressed extensively throughout the nervous system, including sensory, motor, and interneurons. In addition, some neuropeptide genes are also expressed in non-neuronal tissues. In *C. elegans*, neuropeptides function widely in sensations, locomotion, feeding, dauer formation, mating, egg laying, social behavior, sleep and lethargus, learning and memory, etc (Li and Kim, 2008; Van den Pol, 2012; Bhat et al., 2021). This study indicates that FLP-4 from ADL acts with the NPR-5 receptor to augment acute hyperosmotic response in ASH. ADL expresses both FLP-4 and FLP-21. However, the *flp-21*(*ok889*) displays increased avoidance of the medium hyperosmolality, and the behavioral phenotype cannot be restored to the wild type by the *flp-21* genetic rescue in its expression cells or ADL alone. FLP-21 is a known ligand of the NPR-1 receptor. The function of FLP-21 should be discussed in conjunction with its receptor, NPR-1. NPR-1 signaling in ASH inhibits *C. elegans*’ avoidance of copper ions and hyperosmolality by glycerol under food deprivation (Ezcurra et al., 2016). NPR-1, a known receptor of FLP-18 and FLP-21, controls solitary and social feeding (de Bono and Bargmann, 1998; Rogers et al., 2003). In addition, it regulates locomotion based on the following facts. The loss-of-function mutation of *flp-18* and *flp-21* results in increased swimming rates (Chang et al., 2015), and FLP-18 controls the reversal length by acting with NPR-4 on AVA interneurons and NPR-1 on ASE presynaptic to AIY, AIA, and AIB (Bhardwaj et al., 2018). It sounds reasonable that FLP-21 signaling does not engage in ADL exciting ASH in the hyperosmotic avoidance behavior. One explanation of the behavioral phenotype in the *flp-21*(*ok889*) that was unable to be rescued by *flp-21* reexpression is that only one strain was used in this study. In addition, mutation of other gene/s in these mutant animals should not be excluded. The background gene mutation may also explain varied dauer entry in *C. elegans* strains of *flp-21*(*ok889*), *flp-21*(*pk1601*), and *flp-21*(*sy880*) display, among them, only *flp-21*(*pk1601*) showed non-WT phenotype (Lee et al., 2017).

Biogenic amines tyramine and octopamine are invertebrate neurotransmitters analogous to vertebrate epinephrine and norepinephrine, respectively. These monoamines are synthesized from the same precursor, the amino acid tyrosine. Two enzymes catalyze the biosynthesis processes. Tyrosine decarboxylase decarboxylates tyrosine to produce tyramine. Tyramine beta hydroxylase hydroxylates tyramine to form octopamine. Tyramine not only acts as the biological precursor of octopamine. Both compounds are independent neurotransmitters that act through G protein-coupled receptors (Roeder, 2005). The functions of octopamine are recognized better than those of tyramine. Octopamine involves in arousal mechanisms of the visual pathway in lotus (Bacon et al., 1995), the fight or flight response (Adamo et al., 1995), and wake-promotion in *Drosophila* (Crocker and Sehgal, 2008; Crocker et al., 2010). It depresses egg-laying and food-stimulated pharyngeal pumping in *C. elegans* (Horvitz et al., 1982) and acts as a neuromodulator to induce associative odor learning in honeybees (Hammer and Menzel, 1998). Octopamine/G_q_ signaling mediates the activation of cAMP response element-binding protein-dependent gene expression in *C. elegans*, a metabolic adaptation to starvation (Suo et al., 2006). Tyramine is an essential learning cue in *C. elegans* (Jin et al., 2016). Tyramine/TYRA-2 and PDF (arthropod Pigment Dispersing Factor)-2/PDFR (PDF Receptor)-1 signaling pathways set the decision balance between retreat from an osmotic threat and approach to food odor (Ghosh et al., 2016). Tyramine and octopamine receptor TYRA-3 are involved in *C. elegans*’ decision-making (Bendesky et al., 2011).

Octopamine and tyramine play antagonistic roles in regulating multiple physiological processes and behaviors. In *Drosophila* larval, octopamine enhances locomotion during states of hunger, whereas tyramine reduces locomotion during satiation, and a balance between the two is vital in producing normal behavior (Saraswati et al., 2004; Fox et al., 2006; Koon et al., 2011; Schutzler et al., 2019). In the honeybee, octopamine increases, and tyramine decreases eye photo-response (Schilcher et al., 2021). This study indicates that octopamine (from RIC) acting on AIY *via* OCTR-1 signaling enhances, and tyramine (from RIM) downregulates by suppressing ADL hyperosmotic response through TYRA-3 signaling, avoidance of mild and medium osmolality in *C. elegans*. Interestingly, in addition to the action on AIY, octopamine functions as an autocrine signal to auto-excite *via* SER-6 signaling.

Almost every sense organ, including the acoustic, visual, olfactory, tactile, gustatory, and proprioceptive, is modulated by octopamine. However, it is unknown whether other sense organs are modulated by tyramine (Roeder, 2005). Tyramine upregulates ASH Ca^2+^ responses to 3 M fructose (Ghosh et al., 2016) in *C. elegans*. This study indicates that tyramine from RIM interneurons acts as a paracrine signal to inhibit osmosensory output in ADL sensory neurons in the nematode.

The external or internal osmolality may ultimately change cellular osmolality, volume, and stress (Bourque, 2008). Mechanosensitive TRPV channels act as osmosensory transducers (Oliet and Bourque, 1993; Colbert et al., 1997; Liedtke et al., 2000, 2003; Liedtke, 2006, 2007; Moore and Liedtke, 2017). In *C. elegans*, OSM-9, a member of the TRPV family, is involved in osmosensation (Colbert et al., 1997; Liedtke et al., 2003). The *trpv* genes are expressed in multiple types of neurons. Osmosensitive neurons may not be limited to a specific type of sensory neuron. Our preliminary identification of potential osmosensitive neurons, which was focused on glutamatergic neurons, suggests that ASH, ADL, ASE, ASG, AWC, AQR, and PQR, are possibly involved in osmoavoidance and thus may be osmosensitive. AQR and PQR were known to regulate the increased turning rate in response to the osmotic upshift *via* the signaling of cGMP-gated sensory channel subunit TAX-2 (Yu et al., 2017). Both neurons express *tax-2*, *tax-4*, and multiple *gcy* (Guanylyl Cyclase) genes: *gcy-25*, *gcy-32*, *gcy-33*, *gcy-34*, *gcy-35*, *gcy-36*, and *gcy-37*. Both neurons may sense hyperosmolality by guanylyl cyclases—cGMP—TAX-2/TAX-4 signaling. However, molecular mechanisms of hyperosmotic sense in these neurons need further study. Among the known and potential hyperosmolality-sensitive neurons, ASH, ADL, ASE, ASG, and AWC express *osm* (OSMotic avoidance abnormal)-9 encoding a homolog of the mammalian TRPV channel OSM-9, which acts in the detection of strong external osmotic shocks. These sensory neurons have chemo-or/and electro-synaptic connections with interneurons or/and motor neurons, such as AVA, AVB, AVD, AVE, AIA, AIB, AIZ, and RIA. These neurons control or regulate animals’ locomotion (Tsalik and Hobert, 2003; Wakabayashi et al., 2004; Gray et al., 2005; Piggott et al., 2011; Pokala et al., 2014). AIY interneurons suppress turns and reversals and enhance smooth forward movements and dispersal (Tsalik and Hobert, 2003; Wakabayashi et al., 2004; Gray et al., 2005; Li et al., 2014). However, in this study’s wet drop test of medium hyperosmolality, AIY is required for normal hyperosmotic avoidance in *C. elegans*. This neuron functions to upregulate hyperosmotic avoidance. The different AIY roles in varied modalities of behaviors may reflect the complexity of AIY functions and its dynamic regulation of activities by multiple inputs.

## Materials and methods

### *Caenorhabditis elegans* strains

Animals of all *C. elegans* strains were cultured on nematode growth media (NGM) plates at 20°C using *E. coli* bacteria OP50 as food by standard procedures (Brenner, 1974). The strains were obtained from the CGC^5^ or the National Bio-Resources Project.^6^ All transgenic animals were generated with standard microinjection techniques (Mello et al., 1991). The injection pressure was controlled by a DMP-300 digital pneumatic microinjection pump (Micrology Precision Instruments, Ltd, Wuhan, China). Plasmids were injected at 50 ng/μL together with *lin-44p*::*GFP* as a coinjection marker at 10 ng/μL. All strains used in this study are listed in Supplementary Table S1. We used two or three lines of transgenic worms to conduct experiments and summarized all data for statistical analysis.

### Molecular biology

All expression constructs were generated with a Three-Fragment Multisite Gateway® system (Invitrogen™, Thermo Fisher Scientific, Waltham, MA, United States). Briefly, three entry clones comprising three PCR products (promoter, the gene of interest, and *sl2e*::*TagRFP-t, sl2d*::*GFP,* or *unc-54* 3’ UTR, in the name of slot1, slot2, and slot3, respectively) were recombined into the pDEST™ R4-R3 Vector II or custom-modified destination vectors using *att*L-*att*R (LR) recombination reactions to generate expression clones.

We constructed an “A” entry clone containing a sequence of promoters used in this study by the In-Fusion method. In short, a modified *att*L4-*att*R1 entry clone was linearized by PCR. Then, the linearized product and a promoter PCR product were used to generate an “A” entry clone using the ClonExpress®II One Step Cloning Kit (Vazyme Biotech Co., Ltd., Nanjing, China). The PCR products of promoters were amplified from *C. elegans* genomic DNA with primers containing 15–20 bp sequences that carry some sequence of *att*L4 and *att*R1 recombination sites. Alternatively, promoter vectors in the *C. elegans* Promoters Library (Thermo Fisher Scientific, Waltham, MA, United States) were directly used. The length of each promoter used in this study is as follows: *ver-2p* 2.7 kb (in ADL), *gpa-11p* 3.3 kb (in ASH and ADL), *sra-6p* 3.8 kb (in ASH and ASI), *srv-11p* 1.9 kb (in ASH), *flp-21p* 6.6 kb, *npr-5p* 3.0 kb, *npr-4p* 4.0 kb, *eat-4p* 5.6 kb, *sra-9p* 4 kb (in ASK), *flp-17p* 3.3 kb (in BAG), *srh-11p* 0.7 kb (in ASJ), *srh-142p* 3.5 kb (in ADF), *gcy-32p* 0.8 kb (in AQR, PQR, and URX), *str-1p* 4.0 kb (in AWB), *str-2p* 3.7 kb (in AWC), *srsx-3p* 0.9 kb (in AWB and AWC), *gcy-5p* 3.2 kb (in ASER), *gcy-7p* 1.3 kb (in ASEL), *gcy-15p* 0.8 kb (in ASG), *nmr-2p* 4.9 kb, *gcy-13p* 2.3 kb (in RIM), *tdc-1p* 3.0 kb (in RIM and RIC), *tyra-3p* 4.2 kb, *dat-1p* 0.7 kb (in ADE, CEP, and PDE), *gcy-18p* 0.8 kb (in AFD and AIM), *tbh-1p* 4.5 kb (in RIC), *ser-6p* 3.5 kb, *octr-1p* 3.9 kb, and *ttx-3p* 3.1 kb (in AIY, Hobert et al., 1997), *flp-4p* 3.3 kb, respectively.

BP recombination reactions were used to generate entry clones B (containing a sequence of a tested gene) and C (containing *sl2e*::*TagRFP-t, sl2d*::*GFP,* or *unc-54* 3’ UTR). The following genes were used to create the B entry clones. The *flp-21*, *npr-4*, *npr-5*, *tdc-1, octr-1, and ser-6* genes were amplified from the genomic DNA of wild-type N2 worms. *Nmr-2*, *eat-4*, *tyra-3*, *ser-6*, and *tbh-1* cDNA were amplified by reverse transcription-PCR (RT-PCR) from *C. elegans* mixed-stage RNA. Their PCR products flanked by *att*B1 and *att*B2 were recombined with the pDONR-221 vector containing *att*P1 and *att*P2. To generate entry clone C, the BP reaction sites *att*B2r and *att*B3 were inserted into the sequences of the *sl2e*::*TagRFP-t, sl2d*::*GFP,* or *unc-54* 3’ UTR and recombined with the PDONR-P2R-P3 vector. All primers for cloning these promoters and genes are listed in Supplementary Table S2.

### Behavioral assays

#### Preparation of hyperosmotic solutions

Hyperosmotic solutions were prepared by putting solutes in the M13 buffer. M13 buffer consists of (in mM) Tris 30, NaCl 100, and KCl 10 (pH 7.2, adjusted by 1 M HCl or 1 M NaOH). The solutions’ osmolality was measured using Osmomat-3,000 (Gonotec GmbH, GSG-Hof Reuchlinstr. 10–11, Berlin, Germany). The measured osmolality (in Osm) was: M13 buffer, 0.28; 0.1 M glycerol/M13 0.41; 0.5 M glycerol/M13 0.88; 1 M glycerol/M13 1.37; 2 M glycerol/M13 2.29; 0.25 M NaCl/M13 0.77; 0.5 M fructose/M13 0.87; 0.5 M sorbitol/M13, 0.90.

#### Hyperosmotic shock assay

All experiments were performed using synchronized young adult animals maintained on 6 cm nematode growth medium (NGM) plates and at 20°C. The hyperosmotic shock test was performed using a wet-drop test for assaying avoidance of acute hyperosmotic simulation, as previously described (Hilliard et al., 2004; Guo et al., 2015; Krzyzanowski et al., 2016; Wu et al., 2022). Briefly, 10 or more young adults were transferred onto NGM plates without food, OP50 bacteria lawn, and let freely move to remove bacteria. Ten minutes later, an individual animal was stimulated by a droplet (approximately a few hundred nano-litters) of hyperosmotic solutions applied *via* a glass micropipette onto the tail of the forward-moving worm. All tested animals’ avoidance response to the droplet of hyperosmotic solutions was scored as the percentage of animals that displayed reversal or Ω turn for more than one-half of body length within 4 s.

#### Test of the response to gradual internal osmolality change

A droplet assay was used to examine the response to gradual internal osmolality upshifts, essentially as described (Yu et al., 2017). Shortly, a 6 μL solution droplet was placed on a cover glass. An adult animal was transferred from culture plates into a drop of M13 buffer (about 6 μl) and let freely move for 10 min to remove bacteria. Then, the animal was transferred with an eyebrow into the test droplet. Worm movement in the droplet was recorded under a Zeiss Discovery V8 stereoscope (Carl Zeiss MicroImaging GmbH, Göttingen, Germany) using an Andor iXonEM+ DV885K EMCCD camera (Andor Technology plc., Springvale Business Park, Belfast, United Kingdom) at 10 Hz. A reversal or big body bend identified manually by video replay was counted as a turn.

#### Calcium imaging

Cytosolic calcium transients in the soma of tested neurons were measured by detecting changes in the fluorescence intensities of genetically encoded Ca^2+^ indicators G-CaMP3 or G-CaMP6F. The calcium indicators were excited by 460–470 nm light emitted by an Osram Diamond Dragon LBW5AP light-emitting diode (LED) model (Osram, Marcel-Breuer-Straße 6, Munich, Germany) constructed in a multi-LED light source (MLS102, InBio Life Science Instrument Co. Ltd., Wuhan, China) and filtered with a Semrock FF01-520/35–25 emission filter (IDEX Health & Science, LLC, Oak Harbor, WA, United States). Fluorescence images were captured with an Andor iXon^EM^ + DU885K EMCCD camera with 256 × 256 pixels at 10 frames per second under an Olympus IX-70 inverted microscope (Olympus, Tokyo, Japan) equipped with a 40× objective lens [numerical aperture (NA) = 1.3]. A homemade PDMS microfluidic device was used to trap worms and deliver solutions (Guo et al., 2015; Wang et al., 2016; Liu et al., 2019; Wen et al., 2020; Wu et al., 2022).

We employed Day-1 adult animals for all calcium imaging and used them once. Animals collected from culture plates were put into the M13 buffer to remove bacteria for 10 min before being loaded into the microfluidic chip. Because *C. elegans* worms are sensitive to the blue light used for Ca^2+^ imaging, we exposed the tested animal to fluorescent excitation light for 2 min before recording to decrease the impact of light on Ca^2+^ fluorescence for all Ca^2+^ fluorescence imaging tests. The average fluorescence intensity of the region of interest (ROI) of the tested neuron soma was captured and analyzed using Image-Pro Plus 6.0 (Media Cybernetics Inc., Rockville, MD, United States). A nearby region with an area similar to that of the tested soma was used to measure background signals. The percent change of average fluorescence intensity *ΔF/F_0_* was plotted as a function of time for all curves. Where *ΔF* = *F*﹣*F_0_*; *F*, the average fluorescence intensity of the region of interest (ROI) of neuronal soma in each frame; *F_0_*, the average fluorescence intensity of the ROI within the initial 5 s (in ASH, ADL, RIC, and AIY) or 60 s (in RIM) before application of the hyperosmotic solution. The average background signal was subtracted from *F* and *F_0_*. The Ca^2+^ signals were shown by curves, box plots, and heat maps. Box plots showed analyzed data of the average intensity of Ca^2+^ signals during glycerol/M13 solution perfusion.

#### Genetic manipulation of tested neurons

For chemogenetic silencing of the tested neurons, we used neuron type-specific promoters to drive specific expression of the *Drosophila HisCl1* gene in the tested neurons and employed 10 mM histamine to activate the channels. For the preparation of test agar plates containing 10 mM histamine, a stock solution of histamine (1 M in M13 buffer) was diluted with ultrapure water into a 10 mM working solution, and 10% (v/v) working solution was added to the agar solution at approximately 60°C before making the plates. Tested animals were transferred to the test plate containing 10 mM histamine or no histamine as a control and let them freely move for 10 min before hyperosmotic shock assay. HisCl1 transgenic animals were pretreated for 10 min in M13 buffer without (as controls) or with 10 mM histamine for calcium imaging experiments. HisCl1 channel may have a low probability of opening in the absence of ligand histamine, causing a leaking current in some cases. To reduce the leaky effect on tests, we reduced the injected plasmids containing *HisCl1*.

To genetically block vesicular release in the tested neurons, we employed neuron-specific extrachromosomal expression of *TeTx* to intercept vesicular release. TeTx is a specific protease of synaptobrevin that has been successfully used to block vesicle fusion with presynaptic fusion, thus inhibiting or eliminating chemical or even electrical synaptic transmission of tested neurons in *C. elegans* (Schiavo et al., 1992; Guo et al., 2015; Wang et al., 2016; Liu et al., 2019; Ge et al., 2020; Wen et al., 2020; Wu et al., 2022).

### Data analyses

Data of avoidance ratio are displayed as box plots, with each dot representing the data from each individual tested animal or each test. The Ca^2+^ signal data are expressed as heatmaps, box plots, or as the means ± SEM indicated by solid traces ± gray shading. Data were statistically analyzed using software packages in GraphPad Prism 8 (GraphPad Software, Inc., San Diego, CA, United States). When the comparison was limited to 2 groups, an unpaired *t*-test was used to analyze differences and calculate *p*-values. When more than two groups of data were compared, data were analyzed by ordinary one-way or two-way analysis of variance (ANOVA), with recommended *post hoc* tests in the GraphPad Prism 8 software package. Dunnett’s multiple comparison correction was applied when multiple samples were compared to a single sample, i.e., wild-type N2 or other controls. Tukey’s multiple comparison correction was used when multiple samples were compared. The *p*-value is indicated as follows: ns, not significant, ^*^p < 0.05, ^**^p < 0.01, ^***^p < 0.001, ^****^p < 0.0001, and in different colors for varied comparisons.

## Data availability statement

The original contributions presented in the study are included in the article/Supplementary material, further inquiries can be directed to the corresponding author.

## Author contributions

Z-XW supervised the study. HL, J-JW, and RL performed the major part of the experiments, analyzed the data, and created the figures. P-ZW, J-HH, YX, P-PW, J-LZ, and S-JL performed the minor part of the experiments. HL and Z-XW wrote the paper. All authors contributed to the article and approved the submitted version.

## Funding

This work was supported by grants from the National Natural Science Foundation of China (32071013 and 31471034).

## Conflict of interest

The authors declare that the research was conducted without any commercial or financial relationships that could be construed as a potential conflict of interest.

## Publisher’s note

All claims expressed in this article are solely those of the authors and do not necessarily represent those of their affiliated organizations, or those of the publisher, the editors and the reviewers. Any product that may be evaluated in this article, or claim that may be made by its manufacturer, is not guaranteed or endorsed by the publisher.

## Footnotes

^1^ http://www.wormatlas.org

^2^ https://cengen.shinyapps.io/CengenApp/

^3^ https://wormbase.org/

^4^ http://wormweb.org

^5^ http://www.cbs.umn.edu/CGC/

^6^ http://www.shigen.nig.ac.jp/c.elegans/index.jsp
