# Supplementary material for: Disexcitation in the ASH/RIM/ADL negative feedback circuit fine-tunes hyperosmotic sensation and avoidance in Caenorhabditis elegans
Source: Front Mol Neurosci. 2023 Mar 15;16:1101628. doi: 10.3389/fnmol.2023.1101628 (PMC10050701; doi:10.3389/fnmol.2023.1101628)
Supplement: Supplementary file 1 [file Data_Sheet_1.PDF]

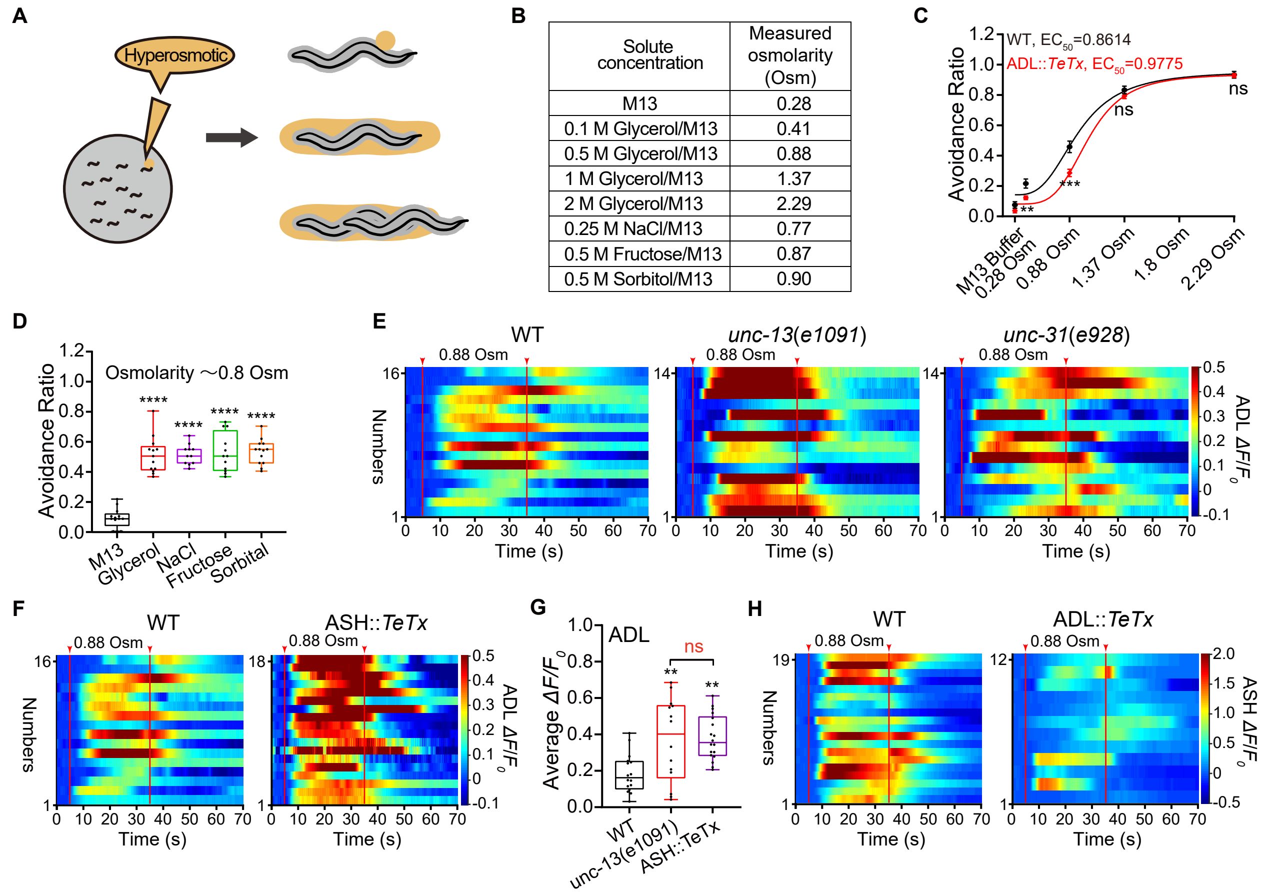

Figure S1. Hyperosmotic avoidance assayed by a wet drop test and somal Ca^{2+} signals shown by heat maps in ASH neurons in response to hyperosmolality. Related to Figure 1.

A. Illustration of a wet drop test with a droplet of hyperosmotic solutions. **B.** Measured osmolarity of solutions indicated. **C.** The animals' avoidance percentage or ratio in response to a droplet of hyperosmotic glycerol/M13 solutions of indicated osmolality in the wet drop test assay. n ≥ 15. **D.** Ratio of avoidance of hyperosmolarity by 0.5 M Glycerol/M13 (0.88 Osm), 0.25 M NaCl/M13 (0.77 Osm), 0.5 M fructose/M13 (0.87 Osm), and 0.5 M sorbitol/M13 (0.90 Osm) solutions in the wild type (WT) N2 animal. **E and F.** Heat maps of percent changes in calcium transients in the soma of ADL sensory neurons in worms of indicated genotypes. Red arrows indicated the time of beginning and ending of the hyperosmolality application. $\Delta F = F - F_0$. F, the average fluorescence intensity in the region of interest (ROI) of neuronal soma in each frame; F_0, that of the soma within 5 s before stimulation. The average background signal was subtracted from all fluorescence. The label on the left *y*-axis indicates the number of tested worms. **G.** Box plots of the average intensity of Ca^{2+} transients of the ON response, with each dot representing the data from each tested animal. **H.** Heat maps of percent changes in calcium transients in the soma of ASH sensory neurons in worms of indicated genotypes. Red arrows indicated the time of beginning and ending of the hyperosmolality application. Statistical significance of difference was analyzed by two-way (in C) or one-way (in D and G) Analysis of Variance (ANOVA) with the post hoc test of Tukey's multiple comparison correction and indicated as follows: ns = not significant, ** $P < 0.01$, *** $P < 0.001$, and **** $P < 0.0001$ in different comparison. In C and G, the tested animal *vs.* WT N2 control, or as indicated; in D, the hyperosmolality treated *vs.* the M13 treated.

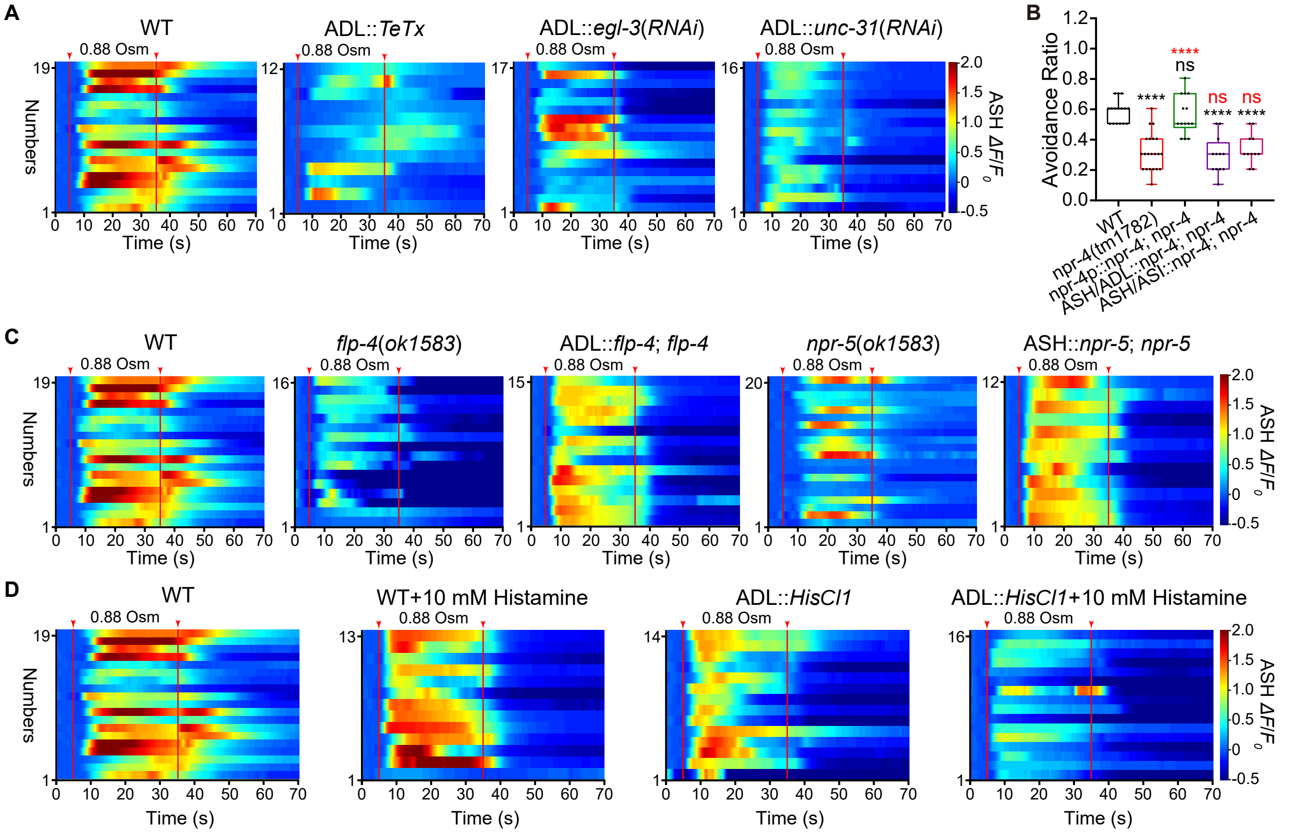

Figure S2. Avoidance of and somal ASH Ca^{2+} responses to medium hyperosmolality of 0.88 Osm in animals of different genotypes. Related to Figure 2.

A. Heat maps of percent changes in calcium transients in the soma of ASH neurons in transgenic worms of genotypes shown. Red arrows mark the time of beginning and ending of the hyperosmolality application. $\Delta F = F - F_0$. *F*, the average fluorescence intensity in the region of interest (ROI) of neuronal soma in each frame. F_0, that of the soma within 5 s before stimulation. The average background signal was subtracted from all fluorescence. The label on the left *y*-axis indicates the number of tested worms. **B.** Ratio of avoidance of the hyperosmolarity (0.88 Osm) stimulus in worms of indicated genotypes. **C and D.** Heat maps of percent changes in calcium transients in the soma of ASH neurons in transgenic worms of genotypes shown. Red arrows mark the time of beginning and ending of the hyperosmolality application. $\Delta F = F - F_0$. *F*, the average fluorescence intensity in the region of interest (ROI) of neuronal soma in each frame. F_0, that of the soma within 5 s before stimulation. The average background signal was subtracted from all fluorescence. The label on the left *y*-axis indicates the number of tested worms.

Statistical significance of difference was analyzed by one-way ANOVA with the post hoc test of Tukey's multiple comparison correction and indicated as follows: ns = not significant, **** $P < 0.0001$, and in different colors for varied comparisons. Black, the tested animals *vs.* wild type (WT) N2 control; red, the gene-rescued *vs.* the related mutant.

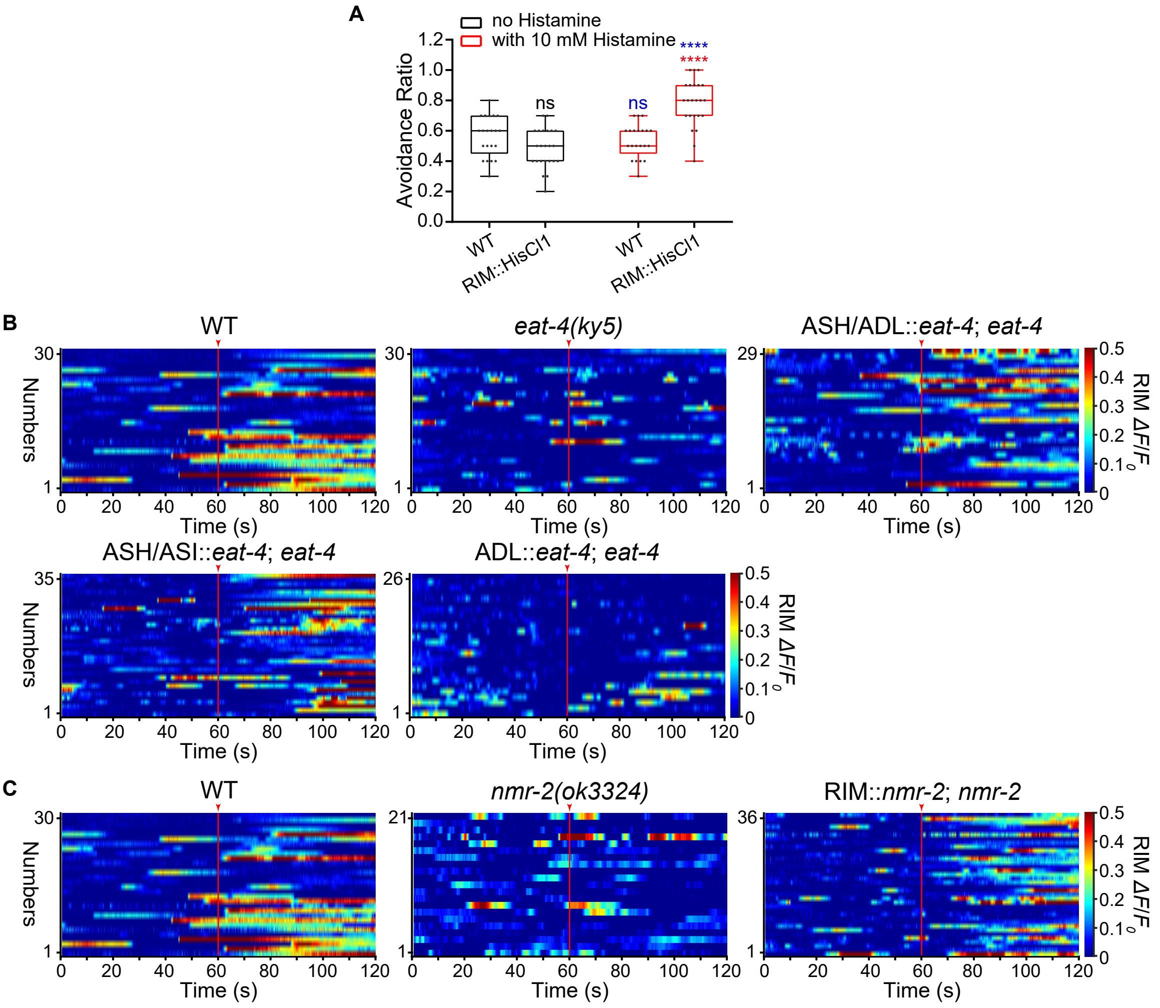

Figure S3. Avoidance of and somal RIM Ca^{2+} responses to hyperosmolality of 0.88 Osm in animals of different genotypes or treatment. Related to Figure 3.

A. Ratio of avoidance of the hyperosmolarity of 0.88 Osm in worms of indicated genotypes. **B and C.** Heat maps of percent changes in calcium transients in the soma of RIM neurons in transgenic animals of genotypes shown. Red arrows mark the time of the beginning of the hyperosmolality application. $\Delta F = F - F_0$. F, the average fluorescence intensity in the region of interest (ROI) of neuronal soma in each frame. F_0, that of the soma within 60 s before stimulation. The average background signal was subtracted from all fluorescence. The label on the left *y*-axis indicates the number of tested worms.

Statistical significance of difference was analyzed by two-way ANOVA with the post hoc test of Tukey's multiple comparison correction and indicated as follows: ns = not significant, **** $P < 0.0001$, and in different colors for varied comparisons. Black or red, the tested animal *vs.* the wild type (WT) N2 control under the condition without or with histamine treatment; blue, the histamine-treated *vs.* the histamine-untreated of the same genotype.

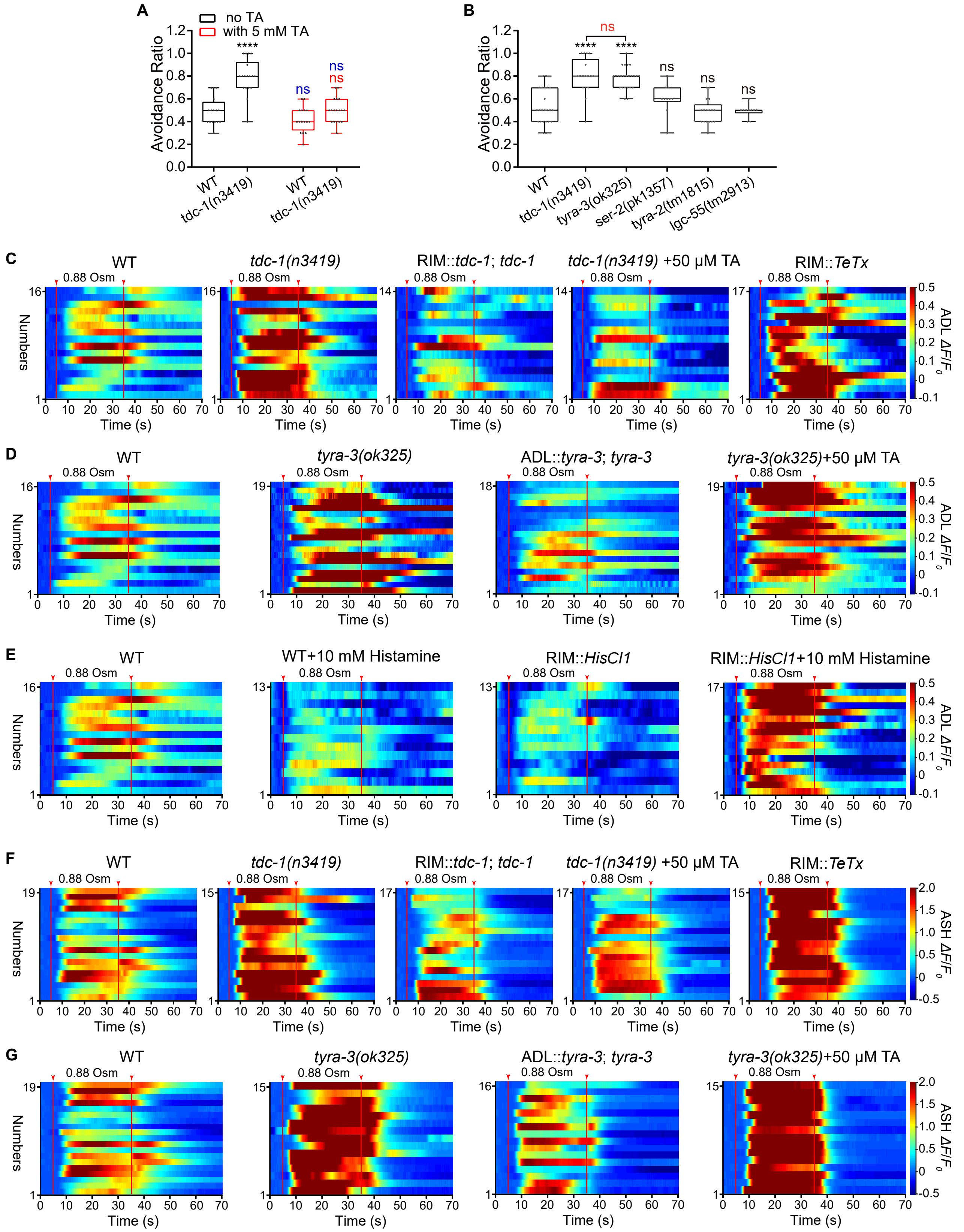

Figure S4. Hyperosmotic avoidance and hyperosmolarity-evoked somal Ca^{2+} signals shown by heat maps in ADL and ASH sensory neurons in animals of different genotypes or tyramine treatment. Related to Figure 4.

A and B. Ratio of avoidance of hyperosmolarity of 0.88 Osm in animals of different genotypes as

indicated. **C - G.** Heat maps of percent changes in calcium transients in the soma of ADL (C - E) and ASH (F and G) neurons in response to 0.88 Osm Glycerol/M13 in transgenic worms of indicated genotypes. Red arrows mark the time of beginning and ending of hyperosmolarity application. $\Delta F = F - F_0$. F, the averaged fluorescence intensity in the region of interest (ROI) of neuronal soma in each frame; F_0, that of the soma within 5 s before stimulation. The average background signal was subtracted from all fluorescence. The label on the left *y*-axis indicates the number of tested worms.
Statistical significance of difference was analyzed by two-way (in A) or one-way (in B) ANOVA with the post hoc test of Tukey's multiple comparison correction and indicated as follows: ns = not significant, **** $P < 0.0001$, in different colors for varied comparisons. In A, black or red, the tested animal *vs.* the wild type (WT) N2 control under the condition without or with tyramine (TA) treatment; blue, the TA-treated *vs.* the TA-untreated of the same genotype. In B, black, the tested animal *vs.* the WT control; red, as indicated.

A

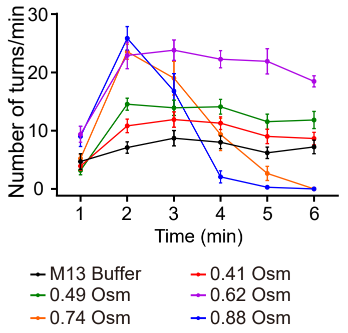

B

	1 min	2 min	3 min	4 min	5min	6 min
WT-M13 Buffer		ns	ns	ns	ns	ns
WT-0.41 Osm	ns	ns / ***	ns / ****	ns / ***	ns / *	ns / *
WT-0.49 Osm	ns	* / ***	ns / ***	ns / ***	ns / ***	* / ****
WT-0.62 Osm	ns	**** / ****	*** / ****	**** / ****	**** / ****	**** / **
WT-0.74 Osm	ns	**** / ****	* / ****	ns / ns	ns / ns	**** / ns
WT-0.88 Osm	ns	**** / ****	ns / *	**** / ns	* / **	**** / **

C

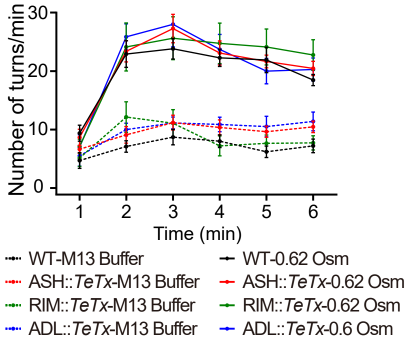

D

	1 min	2 min	3 min	4 min	5min	6 min
WT-M13 Buffer		ns	ns	ns	ns	ns
ASH::*TeTx*-M13 Buffer	ns	ns / ns	ns / ns	ns / ns	ns / ns	ns / ns
RIM::*TeTx*-M13 Buffer	ns	ns / ns	ns / ns	ns / ns	ns / ns	ns / ns
ADL::*TeTx*-M13 Buffer	ns	ns / ns	ns / ns	ns / ns	ns / ns	ns / ns
WT-0.62 Osm		****	****	****	****	**
ASH::*TeTx*-0.62 Osm	ns	ns / ****	ns / ****	ns / ****	ns / ****	ns / ***
RIM::*TeTx*-0.62 Osm	ns	ns / **	ns / **	ns / **	ns / **	ns / *
ADL::*TeTx*-0.62 Osm	ns	ns / ***	ns / ****	ns / ***	ns / *	ns / **

Figure S5. The ASH/RIM/ADL circuit is not involved in aversive behavior response to the upshift of internal osmolality.

A. Curves of the body turn number in the wild type (WT) N2 animal immersed in a drop (about 6 μl) of solutions with varied osmolality as indicated. **B.** Statistical significance of difference indicated in different colors. Red, the hyperosmolality-treated *vs.* the M13-treated; black, different treatment duration *vs.* 1 min. n ≥ 8. **C.** Curves of the body turn number in animals of indicated genotypes in M13-Buffer or solutions of indicated osmolality. **D.** Statistical significance of difference indicated in different colors. Red, the tested animal of indicated genotype *vs.* the WT; black, different treatment duration *vs.* 1 min. n ≥ 9.

Statistical significance of difference was analyzed by two-way ANOVA with the post hoc test of Tukey’s multiple comparison correction and indicated as follows: ns = not significant, * $P < 0.05$, ** $P < 0.01$, *** $P < 0.001$, and **** $P < 0.0001$.

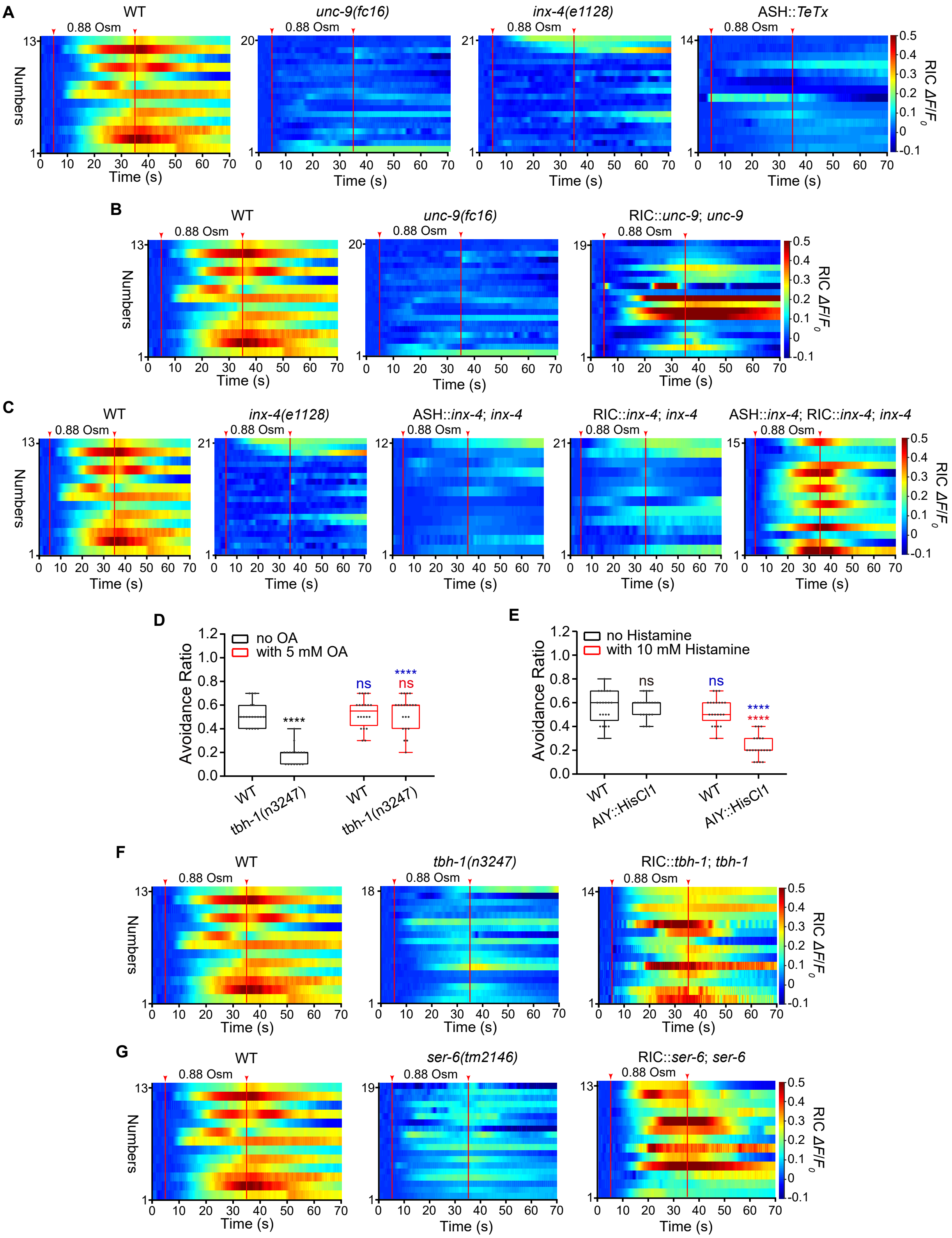

Figure S6. Hyperosmotic avoidance and hyperosmolarity-evoked RIC somal Ca^{2+} signals in animals of different genotypes. Related to Figure 5.

A - C. Heat maps of percent changes in calcium transients in the soma of RIC interneurons in transgenic worms of indicated genotypes. Red arrows mark the time of beginning and ending of the hyperosmolality application. $\Delta F = F - F_0$. F, the average fluorescence intensity in the region of interest (ROI) of neuronal soma in each frame; F_0, that of the soma within 5 s before stimulation. The average

background signal was subtracted from all fluorescence. The label on the left *y*-axis indicates the number of tested worms. **D and E.** Ratio of avoidance of the hyperosmolarity stimulus in worms of different genotypes as shown. **F and G.** Heat maps of percent changes in calcium transients in the soma of RIC interneurons in transgenic worms of indicated genotypes. Red arrows mark the time of beginning and ending of the hyperosmolality application.

Statistical significance of difference was analyzed by two-way ANOVA with the post hoc test of Tukey's multiple comparison correction and indicated as follows: ns = not significant and **** $P < 0.0001$, and in different colors for varied comparisons. Black or red, the tested animal *vs.* the wild type (WT) N2 control under the condition without or with octopamine/histamine-treatment; blue, the octopamine/histamine-treated *vs.* the octopamine/histamine-untreated of the same genotype.

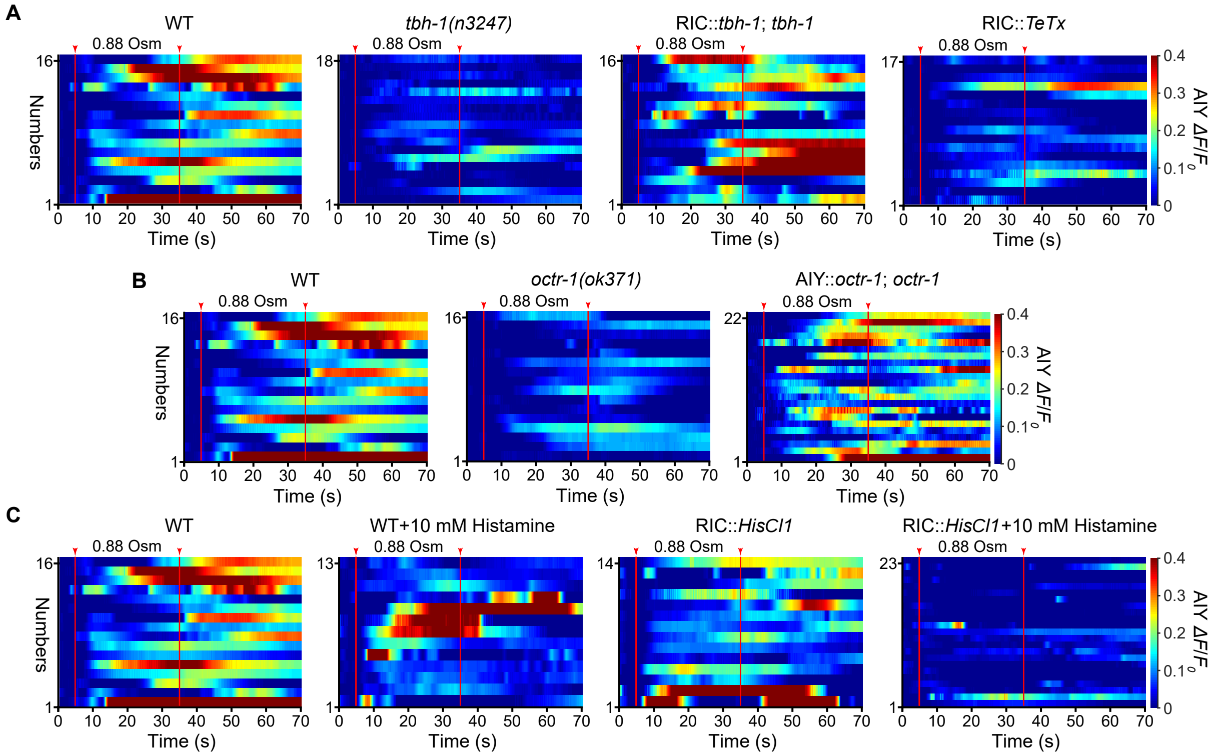

Figure S7. Hyperosmolarity-evoked somal Ca^{2+} signals shown by heat maps in AIY interneurons in animals of different genotypes. Related to Figure 5.

Red arrows mark the time of beginning and ending of the hyperosmolarity application. $\Delta F = F - F_0$. F, the average fluorescence intensity in the region of interest (ROI) of neuronal soma in each frame; F_0, that of the soma within 5 s before stimulation. The average background signal was subtracted from all fluorescence. The label on the left *y*-axis indicates the number of tested animals.

Table S1. The *C. elegans* strains used in this study. Related to all figures.

Strain	Genotype
N2 Bristol	Wild-type
CB1091	*unc-13*(*e1091*) I
DA509	*unc-31*(*e928*) IV
tm2990	*nlp-7*(*tm2990*) X
VC1399	*nlp-8*(*ok1799*) I
tm6232	*nlp-10*(*tm6232*) III
RB982	*flp-21*(*ok889*) V
PS9050	*flp-4*(*sy1606*) II
tm1782	*npr-4*(*tm1782*) X
RB1393	*npr-5*(*ok1583*) V
tm1888	*ins-1*(*tm1888*) IV
RB2030	*nlp-3*(*ok2688*) X
VC1063	*nlp-15*(*ok1512*) I
MT6308	*eat-4*(*ky5*) III
KP4	*glr-1*(*n2461*) III
RB1808	*glr-2*(*ok2342*) III
tm6403	*glr-3*(*tm6403*) I
tm3239	*glr-4*(*tm3239*) II
tm3506	*glr-5*(*tm3506*) V
tm2729	*glr-6*(*tm2729*) X
tm1842	*glr-7*(*tm1842*) III
VM487	*nmr-1*(*ak4*) II
VC2623	*nmr-2*(*ok3324*) V
tm3785	*nmr-2*(*tm3785*) V
tm1811	*mgl-1*(*tm1811*) X
MT13113	*tdc-1*(*n3419*) II
VC125	*tyra-3*(*ok325*) X
OH313	*ser-2*(*pk1357*) X
tm1815	*tyra-2*(*tm1815*) X
tm2913	*lgc-55*(*tm2913*) V
CW129	*unc-9*(*fc16*) X
CB1128	*Inx-4*(*e1128*) V
MT9455	*tbh-1*(*n3247*) X
tm2104	*ser-6*(*tm2104*) IV
tm2146	*ser-6*(*tm2146*) IV
VC224	*octr-1*(*ok371*) X
RB1622	*ser-3*(*ok1995*) I
ZXW2200	*hkdEx2200*[*ver-2p*::*TeTx*::*sl2e*::*TagRFP-t*; *lin-44p*::*GFP*]
ZXW2201	*hkdEx2201*[*ver-2p*::*HisCl*::*sl2e*::*TagRFP-t*; *lin-44p*::*GFP*]
ZXW2202	*hkdEx2202*[*ver-2p*::*GCaMP3.0*::*unc-54*::*3'UTR*; *lin-44p*::*GFP*]

ZXW2203	*hkdEx2203*[*ver-2p*::*GCaMP3.0*::*unc-54*::*3'UTR*; *lin-44p*::*GFP*]; *unc-13*(*e1091*) I
ZXW2204	*hkdEx*2204[*ver-2p*::*GCaMP3.0*::*unc-54*::*3'UTR*; *lin-44p*::*GFP*]; *unc-31*(*e928*) IV
ZXW2208	*hkdEx*2208[*gpa-11p*::*TeTx*::*sl2e*::*TagRFP-t*; *lin-44p*::*GFP*]
ZXW2210	*hkdEx*2210[*gpa-11p*::*GCaMP3.0*::*unc-54*::*3'UTR*; *lin-44p*::*GFP*]
ZXW2211	*hkdEx*2211[*ver-2p*::*TeTx*::*sl2e*::*TagRFP-t*; *gpa-11p*::*GCaMP3.0*::*unc-54*::*3'UTR*; *lin-44p*::*GFP*]
ZXW2212	*hkdEx*2212[*ver-2p*::*unc-31*(*RNAi*)::*sl2e*::*TagRFP-t*; *lin-44p*::*GFP*]
ZXW2213	*hkdEx*2213[*ver-2p*::*egl-3*(*RNAi*)::*sl2e*::*TagRFP-t*; *lin-44p*::*GFP*]
ZXW2214	*hkdEx*2214[*ver-2p*::*eat-4*(*RNAi*)::*sl2e*::*TagRFP-t*; *lin-44p*::*GFP*]
ZXW2215	*hkdEx*2215[*ver-2p*::*egl-3*(*RNAi*)::*sl2e*::*TagRFP-t*; *gpa-11p*::*GCaMP3.0*::*unc-54*::*3'UTR*; *lin-44p*::*GFP*]
ZXW2216	*hkdEx2216*[*flp-21p*::*flp-21*::*sl2e*::*TagRFP-t*; *lin-44p*::*GFP*]; *flp-21*(*ok889*) V
ZXW2217	*hkdEx2217*[*ver-2p*::*flp-21*::*sl2e*::*TagRFP-t*; *lin-44p*::*GFP*]; *flp-21*(*ok889*) V
ZXW2219	*hkdEx*2219[*npr-5p*::*npr-5*::*sl2e*::*TagRFP-t*; *lin-44p*::*GFP*]; *npr-5*(*ok1583*) V
ZXW2225	*hkdEx*2225[*npr-4p*::*npr-4*::*sl2e*::*TagRFP-t*; *lin-44p*::*GFP*]; *npr-4*(*tm1782*) X
ZXW2226	*hkdEx*2226[*gpa-11p*::*npr-4*::*sl2e*::*TagRFP-t*; *lin-44p*::*GFP*]; *npr-4*(*tm1782*) X
ZXW2227	*hkdEx*2227[*sra-6p*::*npr-4*::*sl2e*::*TagRFP-t*; *lin-44p*::*GFP*]; *npr-4*(*tm1782*) X
ZXW2228	*hkdEx*2228[*gpa-11p*::*GCaMP3.0*::*unc-54*::*3'UTR*; *lin-44p*::*GFP*]; *npr-5*(*ok1583*) V
ZXW2232	*hkdEx2232*[*sra-6p*::*flp-21*::*sl2e*::*TagRFP-t*; *lin-44p*::*GFP*]; *flp-21*(*ok889*) V
ZXW2233	*hkdEx2233*[*gpa-11p*::*flp-21*::*sl2e*::*TagRFP-t*; *lin-44p*::*GFP*]; *flp-21*(*ok889*) V
ZXW2234	*hkdEx2234*[*eat-4p*::*eat-4*(*cDNA*)::*sl2e*::*TagRFP-t*; *lin-44p*::*GFP*];*eat-4*(*ky5*) III
ZXW2235	*hkdEx2235*[*sra-6p*::*eat-4*(*cDNA*)::*sl2e*::*TagRFP-t*; *lin-44p*::*GFP*];*eat-4*(*ky5*) III
ZXW2236	*hkdEx2236*[*gpa-11p*::*eat-4*(*cDNA*)::*sl2e*::*TagRFP-t*; *lin-44p*::*GFP*];*eat-4*(*ky5*) III
ZXW2237	*hkdEx2237*[*sra-6p*::*eat-4*(*RNAi*)::*sl2e*::*TagRFP-t*; *lin-44p*::*GFP*]
ZXW2238	*hkdEx2238*[*gpa-11p*::*eat-4*(*RNAi*)::*sl2e*::*TagRFP-t*; *lin-44p*::*GFP*]
ZXW2239	*hkdEx*2239[*sra-9p*::*TeTx*::*sl2e*::*TagRFP-t*; *lin-44p*::*GFP*]
ZXW2240	*hkdEx*2240[*flp-17p*::*TeTx*::*sl2e*::*TagRFP-t*; *lin-44p*::*GFP*]
ZXW2241	*hkdEx*2241[*srh-11p*::*TeTx*::*sl2e*::*TagRFP-t*; *lin-44p*::*GFP*]
ZXW2242	*hkdEx*2242[*srh-142p*::*TeTx*::*sl2e*::*TagRFP-t*; *lin-44p*::*GFP*]
ZXW2243	*hkdEx*2243[*gcy-23p*::*TeTx*::*sl2e*::*TagRFP-t*; *lin-44p*::*GFP*]
ZXW2244	*hkdEx*2244[*str-1p*::*TeTx*::*sl2e*::*TagRFP-t*; *lin-44p*::*GFP*]
ZXW2245	*hkdEx*2245[*str-2p*::*TeTx*::*sl2e*::*TagRFP-t*; *lin-44p*::*GFP*]
ZXW2246	*hkdEx*2246[*srsx-3p*::*TeTx*::*sl2e*::*TagRFP-t*; *lin-44p*::*GFP*]
ZXW2247	*hkdEx*2247[*gcy-5p*::*TeTx*::*sl2e*::*TagRFP-t*; *lin-44p*::*GFP*]
ZXW2248	*hkdEx*2248[*gcy-7p*::*TeTx*::*sl2e*::*TagRFP-t*; *lin-44p*::*GFP*]
ZXW2249	*hkdEx*2249[*gcy-15p*::*TeTx*::*sl2e*::*TagRFP-t*; *lin-44p*::*GFP*]
ZXW2250	*hkdEx*2250[*gcy-32p*::*TeTx*::*sl2e*::*TagRFP-t*; *lin-44p*::*GFP*]
ZXW2251	*hkdEx2251*[*gcy-5p*::*eat-4*(*RNAi*)::*sl2e*::*TagRFP-t*; *lin-44p*::*GFP*]
ZXW2252	*hkdEx2252*[*gcy-7p*::*eat-4*(*RNAi*)::*sl2e*::*TagRFP-t*; *lin-44p*::*GFP*]
ZXW2253	*hkdEx2253*[*gcy-15p*::*eat-4*(*RNAi*)::*sl2e*::*TagRFP-t*; *lin-44p*::*GFP*]
ZXW2254	*hkdEx2254*[*str-2p*::*eat-4*(*RNAi*)::*sl2e*::*TagRFP-t*; *lin-44p*::*GFP*]
ZXW2255	*hkdEx2255*[*gcy-32p*::*eat-4*(*RNAi*)::*sl2e*::*TagRFP-t*; *lin-44p*::*GFP*]
ZXW2256	*hkdEx*2256[*nmr-2p*::*nmr-2*::*sl2e*::*TagRFP-t*; *lin-44p*::*GFP*]; *nmr-2*(*ok3324*) V
ZXW2257	*hkdEx*2257[*nmr-2p*::*nmr-2*::*sl2e*::*TagRFP-t*; *lin-44p*::*GFP*]; *nmr-2*(*tm3785*) V
ZXW2258	*hkdEx*2258[*gcy-13p*::*nmr-2*::*sl2e*::*TagRFP-t*; *lin-44p*::*GFP*]; *nmr-2*(*ok3324*) V

ZXW2259	*hkdEx2259[gcy-13p::nmr-2::sl2e::TagRFP-t; lin-44p::GFP]; nmr-2(tm3785)* V
ZXW2260	*hkdEx2260[gcy-13p::TeTx::sl2e::TagRFP-t; lin-44p::GFP]*
ZXW2261	*hkdEx2261[gcy-13p::HisCl::sl2e::TagRFP-t; lin-44p::GFP]*
ZXW2262	*hkdEx2262[gcy-13p::GCaMP6f::unc-54::3'UTR; lin-44p::GFP]*
ZXW2263	*hkdEx2263[gcy-13p::GCaMP6f::unc-54::3'UTR; lin-44p::GFP]; eat-4(ky5)* III
ZXW2264	*hkdEx2264[gpa-11p::eat-4::sl2e::TagRFP-t; gcy13p::GCaMP6f::unc-54::3'UTR; lin-44p::GFP]; eat-4(ky5)* III
ZXW2265	*hkdEx2265[sra-6p::eat-4::sl2e::TagRFP-t; gcy-13p::GCaMP6f::unc-54::3'UTR; lin-44p::GFP]; eat-4(ky5)* III
ZXW2266	*hkdEx2266[ver-2p::eat-4::sl2e::TagRFP-t; gcy-13p::GCaMP6f::unc-54::3'UTR; lin-44p::GFP]; eat-4(ky5)* III
ZXW2267	*hkdEx2267[gcy-13p::GCaMP6f::unc-54::3'UTR; lin-44p::GFP]; nmr-2(ok3324)* V
ZXW2268	*hkdEx2268[gcy-13p::nmr-2::sl2e::TagRFP-t; gcy-13p::GCaMP6f::unc-54::3'UTR; lin-44p::GFP]; nmr-2(ok3324)* V
ZXW2269	*hkdEx2269[tdc-1p::tdc-1::sl2e::TagRFP-t; lin-44p::GFP]; tdc-1(n3419)* II
ZXW2270	*hkdEx2270[gcy-13p::tdc-1::sl2e::TagRFP-t; lin-44p::GFP]; tdc-1(n3419)* II
ZXW2271	*hkdEx2271[gcy-13p::tdc-1(RNAi)::sl2e::TagRFP-t; lin-44p::GFP]*
ZXW2272	*hkdEx2272[tyra-3p::tyra-3::sl2e::TagRFP-t; lin-44p::GFP]; tyra-3(ok325)* X
ZXW2273	*hkdEx2273[ver-2p::tyra-3::sl2e::TagRFP-t; lin-44p::GFP]; tyra-3(ok325)* X
ZXW2274	*hkdEx2274[dat-1p::tyra-3::sl2e::TagRFP-t; lin-44p::GFP]; tyra-3(ok325)* X
ZXW2275	*hkdEx2275[str-2p::tyra-3::sl2e::TagRFP-t; lin-44p::GFP]; tyra-3(ok325)* X
ZXW2276	*hkdEx2276[gcy-18p::tyra-3::sl2e::TagRFP-t; lin-44p::GFP]; tyra-3(ok325)* X
ZXW2277	*hkdEx2277[flp-17p::tyra-3::sl2e::TagRFP-t; lin-44p::GFP]; tyra-3(ok325)* X
ZXW2278	*hkdEx2278[sra-9p::tyra-3::sl2e::TagRFP-t; lin-44p::GFP]; tyra-3(ok325)* X
ZXW2279	*hkdEx2279[ver-2p::GCaMP3.0::unc-54::3'UTR; lin-44p::GFP]; tdc-1(n3419)* II
ZXW2280	*hkdEx2280[gcy-13p::tdc-1::sl2e::TagRFP-t; ver-2p::GCaMP3.0::unc-54::3'UTR; lin-44p::GFP]; tdc-1(n3419)* II
ZXW2281	*hkdEx2281[ver-2p::GCaMP3.0::unc-54::3'UTR; lin-44p::GFP]; tyra-3(ok325)* X
ZXW2282	*hkdEx2282[ver-2p::tyra-3::sl2e::TagRFP-t; ver-2p::GCaMP3.0::unc-54::3'UTR; lin-44p::GFP]; tyra-3(ok325)* X
ZXW2283	*hkdEx2283[gcy-13p::TeTx::sl2e::TagRFP-t; ver-2p::GCaMP3.0::unc-54::3'UTR; lin-44p::GFP]*
ZXW2284	*hkdEx2284[gpa-11p::GCaMP3.0::unc-54::3'UTR; lin-44p::GFP]; tdc-1(n3419)* II
ZXW2285	*hkdEx2285[gcy-13p::tdc-1::sl2e::TagRFP-t; gpa-11p::GCaMP3.0::unc-54::3'UTR; lin-44p::GFP]; tdc-1(n3419)* II
ZXW2286	*hkdEx2286[gpa-11p::GCaMP3.0::unc-54::3'UTR; lin-44p::GFP]; tyra-3(ok325)* X
ZXW2287	*hkdEx2287[ver-2p::tyra-3::sl2e::TagRFP-t; gpa-11p::GCaMP3.0::unc-54::3'UTR; lin-44p::GFP]; tyra-3(ok325)* X
ZXW2288	*hkdEx2288[gcy-13p::TeTx::sl2e::TagRFP-t; gpa-11p::GCaMP3.0::unc-54::3'UTR; lin-44p::GFP]*
ZXW2289	*hkdEx2289[tbh-1p::GCaMP6f::unc-54::3'UTR; lin-44p::GFP]*
ZXW2290	*hkdEx2290[tbh-1p::GCaMP6f::unc-54::3'UTR; lin-44p::GFP]; unc-9(fc16)* X
ZXW2291	*hkdEx2291[tbh-1p::GCaMP6f::unc-54::3'UTR; lin-44p::GFP];inx-4(e1128)* V
ZXW2292	*hkdEx2292[gpa-11p::TeTx::sl2e::TagRFP-t; tbh-1p::GCaMP6f::unc-54::3'UTR; lin-44p::GFP]*
ZXW2293	*hkdEx2293[tbh-1p::tbh-1::sl2e::TagRFP-t; lin-44p::GFP]; tbh-1(n3247)* X
ZXW2294	*hkdEx2294[tbh-1p::tbh-1(RNAi)::sl2e::TagRFP-t; lin-44p::GFP]*
ZXW2295	*hkdEx2295[ser-6p::ser-6::sl2e::TagRFP-t; lin-44p::GFP]; ser-6(tm2146)* IV
ZXW2296	*hkdEx2296[tbh-1p::ser-6::sl2e::TagRFP-t; lin-44p::GFP]; ser-6(tm2146)* IV
ZXW2297	*hkdEx2297[gpa-4p::ser-6::sl2e::TagRFP-t; lin-44p::GFP]; ser-6(tm2146)* IV
ZXW2298	*hkdEx2298[ver-2p::ser-6::sl2e::TagRFP-t; lin-44p::GFP]; ser-6(tm2146)* IV
ZXW2299	*hkdEx2299[str-1p::ser-6::sl2e::TagRFP-t; lin-44p::GFP]; ser-6(tm2146)* IV
ZXW2300	*hkdEx2300[octr-1p::octr-1::sl2e::TagRFP-t; lin-44p::GFP]; octr-1(ok371)* X
ZXW2301	*hkdEx2301[ttx-3p::octr-1::sl2e::TagRFP-t; lin-44p::GFP]; octr-1(ok371)* X

ZXW2302	*hkdEx2302*[*sra-6p*::*octr-1*::*sl2e*::*TagRFP-t*; *lin-44p*::*GFP*]; *octr-1*(*ok371*) X
ZXW2303	*hkdEx2303*[*gpa-4p*::*octr-1*::*sl2e*::*TagRFP-t*; *lin-44p*::*GFP*]; *octr-1*(*ok371*) X
ZXW2304	*hkdEx2304*[*dat-1p*::*octr-1*::*sl2e*::*TagRFP-t*; *lin-44p*::*GFP*]; *octr-1*(*ok371*) X
ZXW2305	*hkdEx*2305[*tbh-1p*::*TeTx*::*sl2e*::*TagRFP-t*; *lin-44p*::*GFP*]
ZXW2306	*hkdEx*2306[*ttx-3p*::*TeTx*::*sl2e*::*TagRFP-t*; *lin-44p*::*GFP*]
ZXW2307	*hkdEx*2307[*tbh-1p*:: *HisCl*::*sl2e*::*TagRFP-t*; *lin-44p*::*GFP*]
ZXW2308	*hkdEx*2308[*ttx-3p*::*HisCl*::*sl2e*::*TagRFP-t*; *lin-44p*::*GFP*]
ZXW2309	*hkdEx2309*[*tbh-1p*::*GCaMP6f*::*unc-54*::*3'UTR*; *lin-44p*::*GFP*]; *tbh-1*(*n3247*) X
ZXW2310	*hkdEx2310*[*tbh-1p*::*tbh-1*::*sl2e*::*TagRFP-t*; *tbh-1p*::*GCaMP6f*::*unc-54*::*3'UTR*; *lin-44p*::*GFP*]; *tbh-1*(*n3247*) X
ZXW2311	*hkdEx2311*[*tbh-1p*::*GCaMP6f*::*unc-54*::*3'UTR*; *lin-44p*::*GFP*]; *ser-6*(*tm2146*) IV
ZXW2312	*hkdEx2312*[*tbh-1p*::*ser-6*::*sl2e*::*TagRFP-t*; *tbh-1p*::*GCaMP6f*::*unc-54*::*3'UTR*; *lin-44p*::*GFP*]; *ser-6*(*tm2146*) IV
ZXW2313	*hkdEx2213*[*ttx-3p*::*GCaMP3.0*::*unc-54*::*3'UTR*; *lin-44p*::*GFP*]
ZXW2314	*hkdEx2214*[*ttx-3p*::*GCaMP3.0*::*unc-54*::*3'UTR*; *lin-44p*::*GFP*]; *tbh-1*(*n3247*) X
ZXW2315	*hkdEx2215*[*tbh-1p*::*tbh-1*::*sl2e*::*TagRFP-t*; *ttx-3p*::*GCaMP3.0*::*unc-54*::*3'UTR*; *lin-44p*::*GFP*]; *tbh-1*(*n3247*) X
ZXW2316	*hkdEx2216*[*ttx-3p*::*GCaMP3.0*::*unc-54*::*3'UTR*; *lin-44p*::*GFP*]; *octr-1*(*ok371*) X
ZXW2317	*hkdEx2217*[*ttx-3p*::*octr-1*::*sl2e*::*TagRFP-t*; *ttx-3p*::*GCaMP3.0*::*unc-54*::*3'UTR*; *lin-44p*::*GFP*]; *octr-1*(*ok371*) X
ZXW2318	*hkdEx2218*[*tbh-1p*::*TeTx*::*sl2e*::*TagRFP-t*; *ttx-3p*::*GCaMP3.0*::*unc-54*::*3'UTR*; *lin-44p*::*GFP*]
ZXW2319	*flp-4*(*sy1606*); *npr-5*(*ok1583*)
ZXW2320	*hkdEx2320*[*srv-11p*::*TeTx*::*sl2e*::*TagRFP-t*; *ver-2p*::*GCaMP3.0*::*unc-54*::*3'UTR*; *lin-44p*::*GFP*]
ZXW2321	*hkdEx2321*[*srv-11p*::*TeTx*::*sl2e*::*TagRFP-t*; *lin-44p*::*GFP*]
ZXW2322	*hkdEx2215*[*ver-2p*::*unc-31*(*RNAi*)::*sl2e*::*TagRFP-t*; *gpa-11p*::*GCaMP3.0*::*unc-54*::*3'UTR*; *lin-44p*::*GFP*]
ZXW2323	*hkdEx2323*[*flp-4p*::*flp-4*::*sl2e*::*TagRFP-t; lin-44p*::*GFP*]; *flp-4*(*sy1606*) II
ZXW2324	*hkdEx2324*[*ver-2p*::*flp-4*::*sl2e*::*TagRFP-t; lin-44p*::*GFP*] ; *flp-4*(*sy1606*) II
ZXW2325	*h*kd*Ex2325*[*srv-11p*::*npr-5*::*sl2e*::*TagRFP-t; lin-44p*::*GFP*] ; *npr-5*(*ok1583*) V
ZXW2326	*hkdEx2326*[*srv-11p*::*npr-5*::*sl2e*::*TagRFP-t; gpa-11p*::*GCaMP3.0*::*unc-54*::*3'UTR; lin-44p*::*GFP*] ; *npr-5*(*ok1583*) V
ZXW2327	*hkdEx2327*[*srv-11p*::*eat-4*(*RNAi*)::*sl2e*::*TagRFP-t*; *lin-44p*::*GFP*]
ZXW2328	*hkdEx2328*[*ver-2p*::*HisCl1*::*sl2e*::*TagRFP-t; gpa-11p*::*GCaMP3.0*::*unc-54*::*3'UTR; lin-44p*::*GFP*]
ZXW2329	*hkdEx2329*[*gcy-13p*::*HisCl1*::*sl2e*::*TagRFP-t; ver-2p*::*GCaMP3.0*::*unc-54*::*3'UTR; lin-44p*::*GFP*]
ZXW2330	*hkdEx2330*[*tbh-1p*::*unc-9*:: *sl2e*::*TagRFP-t; tbh-1p*::*GCaMP6f*::*unc-54*::*3'UTR; lin-44p*::*GFP*]; *unc-9*(*fc16*) X
ZXW2331	*hkdEx2331*[*srv-11p*::*inx-4*: *sl2e*::*TagRFP-t; tbh-1p*::*GCaMP6f*::*unc-54*::*3'UTR; lin-44p*::*GFP*]; *inx-4*(*e1128*) V
ZXW2332	*hkdEx2332*[*tbh-1p*::*inx-4*: *sl2e*::*TagRFP-t; tbh-1p*::*GCaMP6f*::*unc-54*::*3'UTR; lin-44p*::*GFP*]; *inx-4*(*e1128*) V
ZXW2333	*hkdEx2331*[*srv-11p*::*inx-4*: *sl2e*::*TagRFP-t; tbh-1p*::*inx-4*: *sl2e*::*TagRFP-t; tbh-1p*::*GCaMP6f*::*unc-54*::*3'UTR; lin-44p*::*GFP*]; *inx-4*(*e1128*) V
ZXW2334	*hkdEx2334*[*tbh-1p*::*HisCl1*::*sl2e*::*TagRFP-t; ttx-3p*::*GCaMP3.0*::*unc-54*::*3'UTR; lin-44p*::*GFP*]
ZXW2334	*hkdEx2325*[*ver-2p*::*flp-4*::*sl2e*::*TagRFP-t; gpa-11p*::*GCaMP3.0*::*unc-54*::*3'UTR; lin-44p*::*GFP*] ; *flp-4*(*sy1606*) II

Table S2. List of oligonucleotides used in this study. Related to all figures.

Genes	Forward primers	Reverse primers
ver-2p	TAAATAAAACGTAATATATTG	CATGCAATTTAGTTTTTTATAC
gpa-11p	ATTGCCGCCCGCAAAATA	CATTTTTGGCTGAAAATG
sra-6p	ACTGACTGGGCCGGCCCC	GGAGCAGCACAACTTAAA
gpa-4p	TGCGACTTTCGATACGTA	TGTTGAAAAGTGTTCACA
flp-21p	TGAGGTCACGCAACTTGATGATCAT	CTCCAAAATCCAAAAAGTCATTTTC
npr-5p	TCGTTGCAGTCCGTCTGATTTTATTC	GCATCGAAGGTTCTGGAATTGAAAAT
npr-4p	GTACTATAGGTTTTTGAAAA	TCTGAAATAGAAATTAAAAG
eat-4p	TTTTATTAAATGCAAACGGTGTGCA	GGTTTCTGAAAATGATGATGATGAT
sra-9p	ACAACGGGGCAGCTGCAA	GAAATCTTGAAACTGAAA
flp-17p	TTCCTCTGAAATTATAATATGTTTGA	CTGGAAAAATAAAGTTTTGCGGAAAA
srh-11p	TGAACAGTCTTGCCAACAAC	ACGTATGAATAACGGGTGCT
srh-142p	TTCATTTCTCAATTTTCTAGC	CTAGTTTTGGGTACCTCTAT
gcy-32p	TGTTAATACGTCAAGCAACC	TCTATAATACAATCGTGATC
str-1p	TGCTTTATTATGGTATTTGG	CATTAGTCAAATGATATG
str-2p	AGAGACAGGATACAACTGATAA	TTTTATGGATCACGAGTATTCG
srsx-3p	CCAACAACTTATCAGGTCGA	TCCGAAAAAATATGTATTCTT
gcy-5p	TAGACAAGTGTTTTGGCATG	TTTTCATCAGAATAAGTAAT
gcy-7p	CCTCCTTCGAGAAACATCTACTCCG	ATTATTTTCTTATGCTAAACTGGCA
gcy-15p	TCCGCGAAAGATGCCACCTACCTCTT	GAGCCCCATGGCCGCAACTG
nmr-2p	CTTCACTGCTGCTAAATATT	AGTAAAGAAGATGGACGAGT
gcy-13p	GTGGCCACAATGACAATTATC	GTCCTGAAAAATTATTGAAAG
tdc-1p	ATGGTTTACGGGCTTGGCGAG	TTATATTGGGGTTTGCAATC
tyra-3p	TGATCATTAGACTCCTACAACCT	CTCCGTGTAGATTTCTGAGAATAC
dat-1p	CCATGAAATGGAACTTGAAT	GGCTAAAAATTGTTGAGATT
gcy-18p	CCGAAAGAAGTCGAGTTG	CAACATTTTCTGATGCTCCGA
tbh-1p	TATCGATTAATTTTCTCCATC	GCAACGGCACTTCTCATTTTTC
ser-6p	GCCACGTCACATTGTCCCAT	TTTTTGTGGCGGCGAATTTT
octr-1p	ATCACGTCTACATATCTCAATC	TATGTGATGTTATTTGTTCA
ttx-3p	TACTTCTACAACAGAAATGGTG	ATTGGAATTTAGTCCGACGA
Srv-11p	GGATTAATTGAATTGATG	TGACTAAAGATTTCCTGA
flp-4p	AGCATCGTGATCACAAAA	GTCGCTGTAGTTGTCTGA
TeTx	ATGCCGATCACCATCAAC	TAAGCGGTACGGTTGTA
HisCl1	ATGCAAAGCCCAACTAGCAA	TCATAGGAACGTTGTCCA
flp-21(DNA)	ATGCGGCTGTTCATCTTGCT	TTATCCAAATCGGAGAGGAC
eat-4(cDNA)	ATGTCGTCATGGAACGAGG	TCCTACCACTGCTGATAATG
npr-5(DNA)	ATTGCCTGATGCTTACCT	CACGGACACTACTCTACT
npr-4(DNA)	GTCATAATACGGGAGTATAT	CTAAACTTGTTGACACGTAT
nmr-2(cDNA)	ATGAAACACAGAATTTGG	TCAAAGATTGGTTTCGAC
tdc-1(DNA)	ATGGTTTACGGGCTTGGCGAG	TTACGAAACCAATATCTGT
tyra-3(cDNA)	ATGGCGGCGCTTGGCGGCTCGCTTTGGACGA	TTAGAATATTGTCGATTGCTGATG

tbh-1(cDNA)	ATGATGTCGAGTTATGTGATG	TTAGACACGAGAAGTTGAGC
ser-6(cDNA)	ATGTCTAATTTAAGTTGTGCTGCT	ATAAAAATTGAGAAAGAGCC
octr-1(*DNA*)	ATGTGGAACCTTAACTGCAGTGAAA	TCATTTGTAGAACTCCATGAGTGGA
flp-4(*DNA*)	ATGAACGCCTTCTCCTCTTCCCTTA	TTATTTTCCAAAGCGAATGAATGAT
inx-4(*DNA*)	ATGAACAGACGTGGTACCGGTGGAG	CTATACTGCTAAAGGTACATTTTCC
unc-9(*DNA*)	ATGAGTATGCTATTGTATTATTTCG	TCACACGTCGTGCATTTTTCCTTCT
egl-3(*RNA*, sense)	AATGCCGAAGCTTCATACG	TGTGGAGTGTCAATGAGTCC
egl-3(*RNAi*, antisense)	TGTGGAGTGTCAATGAGTCC	AATGCCGAAGCTTCATACG
unc-31(*RNAi*, sense)	CAGAACTCTCTGCGCAAGGAGGA	CTGCTCATTGGTCGGCACCC
unc-31(*RNAi*, antisense)	CTGCTCATTGGTCGGCACCC	CAGAACTCTCTGCGCAAGGA
eat-4 (*RNAi*, sense)	GTTATGGAGCAAACTTGGATCGG	TGGAGCAGCCCATGAGACGT
eat-4 (*RNAi*, antisense)	TGGAGCAGCCCATGAGACGTATG	GTTATGGAGCAAACTTGGAT
flp-4(RNAi*, sense*)	GCTGAGCAAGAAGAGAAG	CGAATCTGATGAAGGTTG
flp-4(*RNAi*, antisense)	CGAATCTGATGAAGGTTG	GCTGAGCAAGAAGAGAAG
tdc-1(*RNAi*, sense)	CTTGATTGGTTCGGAAAA	TGGCGTCCGCTCTCAGAA
tdc-1(*RNAi*, antisense)	TGGCGTCCGCTCTCAGAA	CTTGATTGGTTCGGAAAA
tbh-1(*RNAi*, sense)	CCAGAACTTCACAAAGGA	TATCGTAGACGCATGTGG
tbh-1(*RNAi*, antisense)	TATCGTAGACGCATGTGG	CCAGAACTTCACAAAGGA
GCaMP6f	CGCCACCATGGGTTCTCATC	ATCACCGTCCCCAGCTCCTT
GCaMP3	ATGGGTTCTCATCATCAT	TTACTTCGCTGTCATCAT
